# Redox-Based Defect Detection in Packed DNA: Insights from Hybrid Quantum Mechanical/Molecular Mechanics Molecular Dynamics Simulations

**DOI:** 10.1021/acs.jctc.3c01013

**Published:** 2023-11-14

**Authors:** Murat Kılıç, Polydefkis Diamantis, Sophia K. Johnson, Oliver Toth, Ursula Rothlisberger

**Affiliations:** Laboratory of Computational Chemistry and Biochemistry, Institute of Chemical Sciences and Engineering, École Polytechnique Fédérale de Lausanne (EPFL), CH-1015 Lausanne, Switzerland

## Abstract

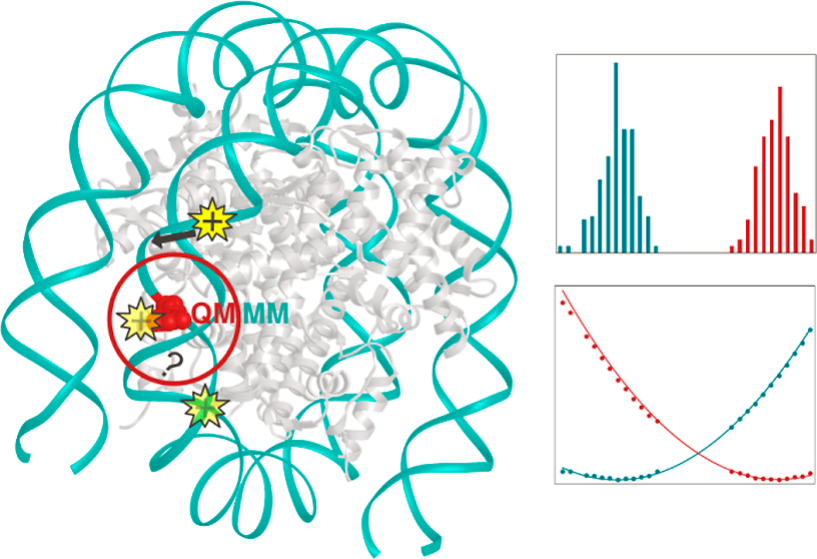

The impact of an 8-oxoguanine (8oxoG) defect on the redox properties of DNA within the nucleosome core particle (NCP) was investigated employing hybrid quantum mechanical/molecular mechanics (QM/MM) molecular dynamics simulations of native and 8oxoG-containing NCP systems with an explicit representation of a biologically relevant environment. Two distinct NCP positions with varying solvent accessibility were considered for 8oxoG insertion. In both cases, it is found that the presence of 8oxoG drastically decreases the redox free energy of oxidation by roughly 1 eV, which is very similar to what was recently reported for free native and 8oxoG-containing DNA. In contrast, the effect of 8oxoG on the reorganization free energy is even smaller for packed DNA (decrease of 0.13 and 0.01 eV for defect-free and defect-containing systems, respectively) compared to the one for free DNA (0.25 eV), consistent with the increased rigidity of the NCP as compared to free DNA. Furthermore, the presence of an 8oxoG defect does not yield any significant changes in the packed DNA structure. Such a conclusion favors the idea that in the case of chromatin, defect-induced changes in DNA redox chemistry can also be exploited to detect damaged bases via DNA-mediated hole transfer.

## Introduction

DNA holds all of the genetic information required for development and survival for every living species and, therefore, is crucially important to protect from damage. Unfortunately, DNA is constantly exposed to harmful sources, such as radiation, reactive oxygen species (ROS), and other chemical agents. In response, our cells developed efficient mechanisms for recognizing and repairing various DNA defects. These mechanisms are of prime significance, as damage to the genome can lead to the development of diseases such as neurodegenerative disorders and cancer.

A substantial fraction of these lesions are defect bases originating from the interaction of DNA with ROS and ionizing radiation^1−3^ and the majority of these concern oxidative derivatives of guanine (G). In fact, among the four native DNA bases, G has the lowest vertical ionization energy (VIE) in gas phase^4^ and in aqueous solution^5,6^ and is thus considered the most readily oxidizable^5,7−9^ and the most common target in this kind of attack. The highly mutagenic 8-oxoguanine (8oxoG) lesion, a product of oxidative damage to G, is indeed the most common of all of the DNA defects. Unlike normal guanine, 8oxoG can more readily form a hydrogen-bonded pair with adenine (A) instead of cytosine (C), resulting in C:G to A:T mutations during replication.^10,11^ Calculations at the single/few base/nucleotide level suggest that 8oxoG has an even lower VIE than G.^12−15^ Moreover, computational investigations showed that G-rich^5,11,15,16^ and 8oxoG-containing^11,15^ fragments of up to 3 base pairs have lower IEs than single G and 8oxoG fragments, consistent with experimental studies,^9,17,18^ indicating that these segments can function as hole sinks by sacrificially attracting oxidative damage, thereby protecting other regions of the DNA. Fragments containing 8oxoG have an especially pronounced potential to act as hole sinks, as evidenced experimentally (by increased strand cleavage at the 8oxoG site following DNA irradiation)^18^ and computationally (by lower IEs for the defect DNA fragments).^12,15^

More broadly, such evidence opens up the possibility that the cell can use local changes in DNA redox chemistry to efficiently detect and repair DNA lesions. Moreover, facile hole and excess electron transport through DNA are well-established and documented phenomena.^19^ Taking this into consideration and based on the conclusions drawn from their experimental investigations, Barton and co-workers have proposed that the recognition of defect bases can occur via a DNA-mediated charge transfer (CT) scheme.^20−25^ Details regarding the mechanism of the suggested CT process have already been extensively discussed.^15,20−26^ The elementary step of this scheme involves a CT process between two base excision repair (BER) enzymes bound to DNA, in which a charge traverses the DNA sequence between them. It is proposed that a defect base will act as a trap for the migrating charge, thereby interrupting the transfer of charge between the two BER enzymes. The two enzymes could consequently scan the DNA sequence between them to identify the presence of a lesion and initiate its repair. A theoretical model predicted that, when compared to the conventionally proposed single BER enzyme scanning, such a CT-based scheme leads to a decrease of the total interrogation time of the entire genome by an order of magnitude.^27^ As already discussed in detail,^26^ evidence of (i) relative differences in ionization energies/electron affinities of small native versus defect fragments of up to three base pairs,^14,15^ and of (ii) the effect of defect bases or base pair mismatches on CT processes in DNA^17,18,28−31^ indicates that hole transfer would be much more likely to be exploited for lesion recognition within a CT mechanism. The possibility of a DNA-mediated hole transfer scheme is further supported by experimental studies for oxidative and bulky lesions utilizing cyclic voltammetry and chronocoulometry.^20^ Ultimately, from an electrochemical perspective, for such a hole-transfer scheme to be efficient, the redox properties of native and damaged DNA need to be substantially different. A relative difference of 0.55 V has been reported between the oxidation potentials (*E*_ox_) of guanosine and 8-oxoguanosine molecules in aqueous solution [−1.29 V versus −0.74 V with respect to the standard hydrogen electrode (SHE)].^17^ However, an overall assessment of the ensemble of values for small fragments shows that the relative redox differences between native and defect-containing systems are not converged up to the largest system sizes considered (ten guanine bases, eight guanine-cytosine base pairs, and six guanine-cytosine nucleotides), with the vertical ionization energy continuing to decrease as the system size is increased.^12−15,17^ In fact, a recent quantum mechanical/molecular mechanics (QM/MM) molecular dynamics (MD) simulation,^26^ in which the redox properties of two extended (39 base pair) DNA fragments, one in its native form and one containing an 8oxoG defect, were computed, demonstrated that the insertion of 8oxoG leads to an increase of the oxidation potential by almost 1 eV. Such a pronounced difference is strongly in favor of a DNA-mediated hole transfer scheme for the recognition of 8oxoG by BER enzymes and underlines the importance of studying extended systems with explicit account of the physiological environment.

While all of the aforementioned investigations do support a DNA-mediated hole transfer scheme for the recognition of 8oxoG and other lesions, they all focus on bare, free DNA systems. However, in the cell, DNA most commonly occurs in a packed form within the chromatin structure. The elementary unit of chromatin is the nucleosome core particle (NCP), consisting of approximately 146 bp wrapped around an octamer of histone proteins. Different NCPs are connected by linker DNA composed of 38–53 base pairs. The structural properties of packed DNA in the NCP differ substantially from those of bare DNA.^32−34^ In view of the fact that in NCPs the electrostatic environment can differ largely from the one of free DNA, with DNA strands (presenting negatively charged phosphodiester backbones) tightly wrapped around highly basic (i.e., positively charged) histone proteins, the same could also hold for the electrochemical properties and thus the redox chemistry, ability to conduct holes of free (unpacked) DNA in aqueous solution, and the potential effect of a defect base could significantly differ when DNA is in its packed state.

Some attempts to investigate DNA-mediated CT in chromatin have been reported in the literature.^35−39^ However, the resulting evidence is contradictory and inconclusive, possibly because of the variety of different donors and acceptors used in the respective experimental setups, making it challenging to directly compare studies and disentangle effects due to the specific redox probes used from the effects due to intrinsic redox features of chromatin. Some of these works also suggest that the efficiency of hole transport and the potential of observing oxidative damage in G-rich sequences depend on the exact position within the NCP with respect to its exposure to the solvent or the histone environment.^35,37,39,40^ Additional investigations, which focus on the potential repair of 8oxoG^41−43^ or a uracil mismatch^44−49^ within the NCP, indicate a decrease in repair efficiency for defects within regions facing toward the interior histone proteins and away from the solvent-facing surface, up to an order of magnitude in the case of uracil mismatch repair. Evidence from many of these studies indicates that BER enzyme binding accessibility is greatly modulated by the local chromatin structure.

As accessibility is hampered for repair enzymes in NCP compared to unraveled DNA, the base-by-base enzyme method of scanning for DNA damage might be less efficient as well. Furthermore, while damages such as double-strand breaks or bulky adducts can cause deformations in DNA helical structure, oxidative lesions tend to be comparatively less disruptive to DNA structure.^50^ However, a study on spiroiminodihydantoin (Sp) suggests that, despite local disruptions to the helix near the Sp-site, NCPs containing Sp display a global structural stability similar to that of NCPs with native DNA.^51^ Such a finding is interesting because the Sp oxidative defect, which arises upon further oxidation of 8oxoG, has an even more distorted shape than its parent 8oxoG base and therefore would be assumed to have a broader impact on NCP formation and unraveling. The exploitation of structural differences alone for the recognition of oxidative lesions might be inefficient if such structural distortions are negligible or highly localized within the NCP.

In the context of the DNA-mediated hole transfer scheme, it is crucial to understand whether defect-induced differences in DNA redox chemistry within the NCP are substantial enough to be leveraged by BER enzymes for the detection of lesions. Such knowledge would help in understanding if the detection of defect DNA bases by BER enzymes could, in principle, also occur within the NCP structure or if—as recently suggested^41−43,52^—it is restricted to processes during which the DNA is found in an unfolded state, such as during replication, which would clearly hamper the overall detection efficiency. Under this scope, we investigate the redox properties of native and 8oxoG-containing DNA in its fully packed form within the NCP using an analogous computational approach as for our recent study of the corresponding redox properties of free DNA.^26^ To this end, we have performed hybrid QM/MM MD simulations of native and 8oxoG-containing NCP systems under biologically relevant conditions, including an explicit representation of water and Na^+^ and Cl^–^ ions at physiological ionic strength. Two different G-rich locations were considered within the NCP, differing in the extent of solvent exposure. For both cases, it is shown that the presence of an 8oxoG decreases the redox potential by approximately 1 eV, suggesting that the defect-induced effect on redox chemistry found in native DNA is present to a similar extent in chromatin.

## Methods

### Theory

The redox properties of the NCP systems were determined employing a theoretical method based on Warshel’s theory of vertical energy gap fluctuations^53,54^ and the general assumption of Marcus theory for electron transfer reactions, while also taking deviations from Marcus theory into consideration.^55−58^ This approach has been extensively employed for the investigation of chemical and biological systems,^59−69^ including the determination of the redox properties of bare DNA fragments in biologically relevant conditions.^26^ Since this method has already been described in detail previously,^26,59,70^ only a brief overview will be presented here.

In short, all of the information needed to compute the redox free energy (Δ*A*_R→O_) and the reorganization free energy (λ) is present in the distributions of the vertical energy gaps (Δ*E*) between the two states. For the reduced (R) and oxidized (O) states, Δ*E* corresponds to the vertical ionization energy (VIE) and electron affinity (VEA), respectively. Two independent MD simulations thus need to be performed for states R and O, from which the VIE and VEA distributions are determined, respectively. Δ*A*_R→O_ can be computed directly from the average ⟨VIE⟩ and ⟨VEA⟩ as follows
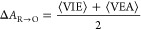
1Within the Marcus theory, VIE and VEA distributions are Gaussian and have equal variances (σ_R_ = σ_O_ = σ). Moreover, given that σ is directly related to λ, the reorganization free energies of the two states are equal as well (λ_R_ = λ_O_ = λ). A single reorganization free energy can thus be defined in association with Δ*A*_R→O_
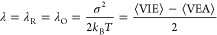
2

Adherence to the assumptions of Marcus theory can then be assessed by comparing the shape and variance of the two Δ*E* distributions as well as by the construction of the Helmholtz free energy curves, which are expected to be intersecting parabolas. The free energy curves can be constructed using the following modified energy gap
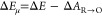
3

In eq 3, – Δ*A*_R→O_ corresponds to the electronic chemical potential of the fictitious electrode, which was selected so that the thermodynamic driving force is zero.

The free energy curves of states R and O are directly related via the following expression
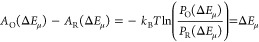
4where *P*_*i*_(Δ*E*_μ_) corresponds to the probability of encountering Δ*E*_μ_ in state *i* (R or O).

### Computational Details

**Figure 1:**
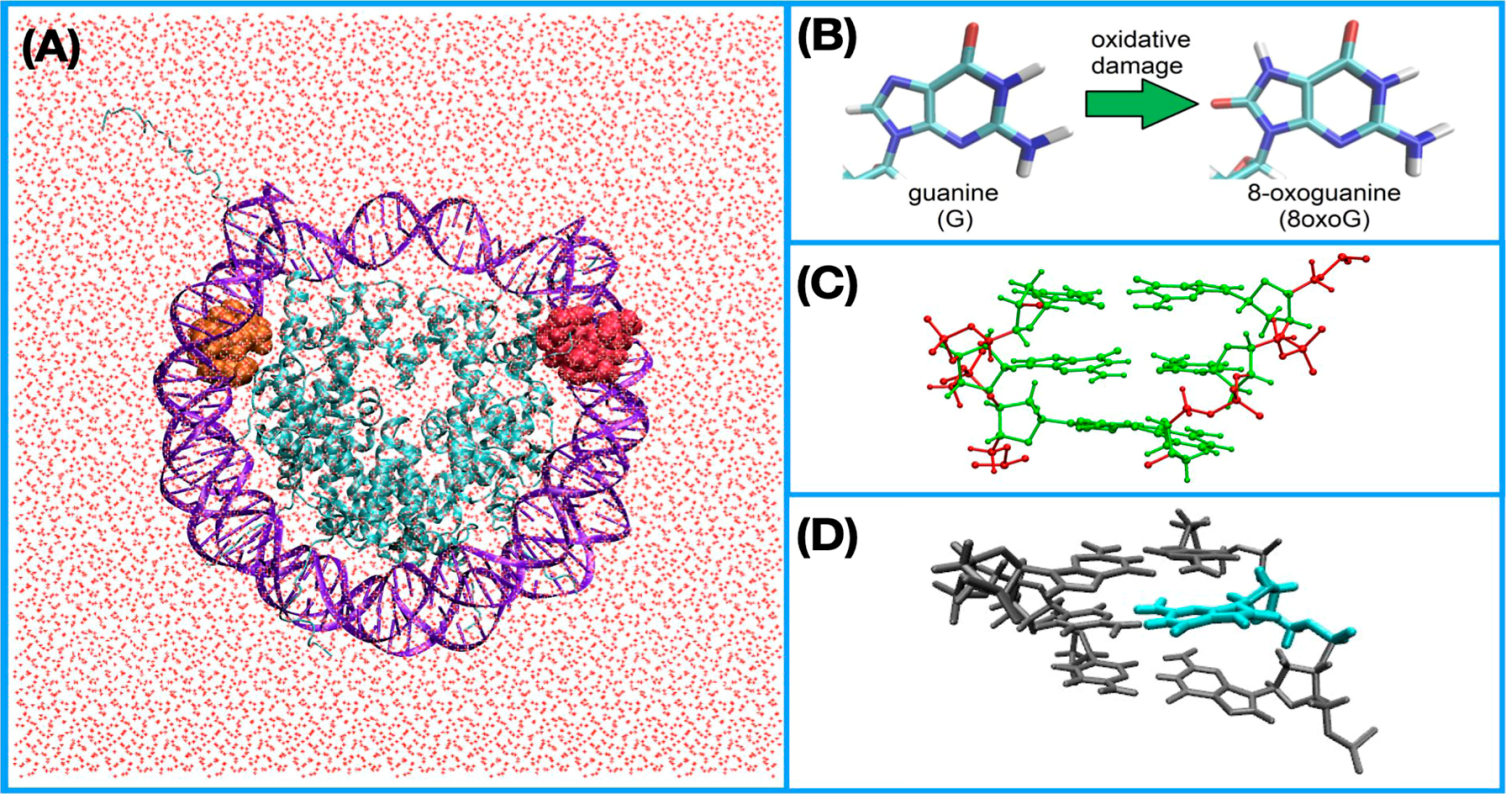
(A) Overview of the periodic box containing the initial nucleosome core particle structure from 1AOI.pdb. The histone octamer is in cyan, the DNA is in magenta, the solvent is shown in a stick representation in the background, and the counterions are not shown. DNA regions shown in red and orange, respectively, correspond to histone-facing and solvent-facing guanine-rich regions 1 and 2. (B) Depiction of guanine and 8-oxoguanine bases. (C) QM/MM partitioning shown with a zoom-in to the guanine-rich region 2, with the atoms treated at the QM and MM levels shown in green and red colors, respectively. (D) Zoom-in to the region of interest for the defect system where 8-oxoguanine (in cyan) was inserted at the G-rich region 1 (other bases in gray).

The nucleosome core particle (NCP) structure was obtained from PDB file 1AOI(71) and was solvated in a periodic orthorhombic box containing approximately 76,500 water molecules (148 Å × 159 Å × 107 Å). The DNA fragment was composed of 146 base pairs. The total charge was neutralized using Na^+^ ions, while additional Na^+^ and Cl^–^ ions corresponding to a biologically relevant concentration of 150 mM^72^ were also added. The simulated systems consisted of approximately 252,465 atoms in total. A visualization of the entire periodically repeated box is shown in Figure 1A.

To assess the effect of specific NCP locations on the change in the DNA redox properties, two different guanine-rich regions were considered. These two regions were the only areas in 1AOI.pdb with three adjacent guanine bases. They comprise residues 58-60/233-235 (5′-GGC-3′/5′-GCC-3′) and 87-89/204-206 (5′-GCC-3′/5′-GGC-3′). In the rest of the manuscript, these two regions will be respectively referred to as region 1 and region 2 (and they are shown in red and orange color in Figure 1A). Both regions were selected within DNA helices firmly wrapped around the histone proteins and not near the more flexible tails. In the respective defect systems, the central G of each region (G59 in region 1 and G205 in region 2) was replaced by an 8oxoG defect (shown in a representative manner for region 2 in Figure 1D). The identity of flanking bases can affect the redox properties of neighboring bases.^73−75^ Of particular interest for this study is that the oxidation potential of guanine is lower when it is found to be flanked by other guanine bases, as in this study’s systems. In our comparison of regions 1 and 2, we separated the influence of the nearby DNA sequence from the possible impact of the rest of the environment (in terms of water accessibility and histone proximity) by making use of a palindromic DNA sequence (see Figure S5 in the Supporting Information), with region 1 and region 2 corresponding to equivalent respective positions (region 1 on strand A and region 2 on strand B), i.e., with the same flanking bases. As a consequence, the two regions studied experience identical neighboring bases, allowing the study to focus on the local environmental impacts on DNA properties, such as solvent accessibility and protein–DNA interactions. Region 1 is found to have more interactions with nearby histone proteins and therefore could be considered less solvent accessible than region 2 (see Tables S3–S4 in the Supporting Information). Furthermore, the three base pairs of region 2 have a greater number of average hydrogen-bonding interactions with solvent molecules than the three base pairs of region 1, which additionally indicates that region 2 is more solvent-exposed than region 1 (see Table S5 in the Supporting Information). Lastly, the central base of the quantum region itself, either guanine or 8oxoG, in region 1 has fewer available hydrogen-bonding solvent partners within 0.35 nm than the central base in the region 2 systems (see Table S6 in the Supporting Information). While the DNA sequences around the two regions studied do not differ, the local environment in terms of solvent accessibility and nearby protein contacts is unique to each region.

The systems were first equilibrated classically using GROMACS 2019.4.^76,77^ The protein and the DNA were described by the AMBER14SB^78^ and parmbsc1^79,80^ force fields, respectively. For 8oxoG, the parameters determined by Miller and co-workers^81^ were employed. For the oxidized state systems, residues 59 (region 1) and 205 (region 2) were modeled using the oxidized state topologies of G/8oxoG (for native/defect systems, respectively) that were employed in our recent investigation of the redox properties of native and defect systems in bare DNA.^26^ Water molecules were modeled with the TIP3P force field,^82^ while Na^+^ and Cl^–^ ions were described with the parameter set developed by Joung and colleagues^83^ (derived for the TIP3P water environment). In total, seven NCP systems were equilibrated classically [native reduced state + (2 × native oxidized state) + (2 × defect reduced state) + (2 × defect oxidized state)]. A time step of 2 fs was employed and bonds to all hydrogen atoms were constrained using the Linear Constraint Solver (LINCS).^84^ A cutoff of 12 Å was used for the real-space part of nonbonded interactions. Long-range electrostatics were treated with the Particle Mesh Ewald (PME) method.^85^ Following an initial energy minimization, the systems were equilibrated in the isothermal–isobaric (*NPT*) and canonical (*NVT*) ensembles for 240 ns and 1 μs, respectively. Temperature and pressure were maintained at 300 K and 1 atm using a Nosé–Hoover thermostat^86^ and Parrinello–Rahman barostat^87,88^ respectively.

DNA structural analysis was performed with the CURVES+ software.^89,90^ This software package is designed to evaluate 41 key structural features of DNA systems, including intrabase and interbase pair parameters as well as groove parameters, among others. The analysis was performed in several parts using approximately 1000 frames sampled at equidistant time intervals from each of the classical trajectories of the reduced wild-type, defect region 1, and defect region 2 systems. A global analysis of 146 base pairs was compared to an analysis in which the first and last 20 base pairs were removed in order to assess the impact of tail flexibility. Then, differences in structural parameter distributions between the wild-type and defect systems were assessed. Finally, when differences between wild-type and defect systems were visually observable in the resulting distributions, a side-by-side comparison for four equally-sized sections of the NCP systems was performed to evaluate how structural differences were localized across the NCP. The four 21 base pair sections of the NCP systems started from the 32nd base pair to the 52nd (Section 1), the 53rd to the 73rd (Section 2 and the region 1 defect section), the 74th to the 94th (Section 3 and the region 2 defect section), and the 95th to the 115th base pair (Section 4). At the classical level, the oxidized and reduced simulations of the same system differ only by one counterion within the entire simulation box, making the reduced and oxidized runs almost equivalent from a classical perspective. With this in mind, we chose to perform the structural analysis on only one of the classical trajectories per system; in this case, we chose reduced systems.

Once the classical equilibration was completed, hybrid QM/MM MD simulations (8 in total) were launched using the QM/MM interface of the CPMD code^91^ with GROMOS,^92,93^ employing the QM/MM coupling scheme developed by Rothlisberger and colleagues.^94−96^ In each case, the three DNA base pairs of interest (residues 58–60/233–235 and 87–89/204–206 for regions 1 and 2, respectively) were treated at the density functional theory (DFT) level, using the BLYP functional for the exchange and correlation energies,^97,98^ in combination with dispersion-corrected atom-centered potentials (DCACPs) to properly account for dispersion forces.^99,100^ The rest of the system was described with the force fields used in classical MD. For each residue of the QM region, the QM-MM boundaries were placed at the C5′–C4′ and C3′–O3′ bonds (as shown in Figure 1C), which were thus capped by dummy hydrogen atoms. Norm-conserving Martins-Troullier pseudopotentials^101^ were employed, and a plane-wave cutoff of 75 Ry was used for the expansion of the Kohn–Sham orbitals. For each system, the first part of the QM/MM protocol consisted of successive equilibrations at 100, 200, and 300 K using Born–Oppenheimer (BO) MD with a time step of 10 atomic units (au), which lasted 20 ps in total. The temperature was maintained using three different Nosé–Hoover thermostats (one for the QM region, one for the MM part of the solute, and one for the solvent and counterions). Following the equilibration, the thermostats were switched off to verify that the system’s energy had stabilized, and a 25 ps-long production phase was carried out with Car–Parrinello (CP) MD,^102^ with a time step of 4 au and a fictitious electron mass of 400 au. The temperature and energy stability of the ensemble were carefully monitored throughout the simulation. The vertical energy gap distributions and redox properties were then calculated as described in the Theory subsection. Based on the convergence of Δ*E* averages and distributions, 1000 frames sampled at equidistant time intervals were analyzed for each trajectory.

Finally, it should be mentioned that, as it has already been discussed in detail,^26,63,66^ redox properties determined with computational schemes such as the one employed here can be subject to corrections to Δ*A*_R→O_ and λ in order to account for contributions due to the finite size of the simulated periodic box and/or to background charge interactions between periodic images when explicit counterions are not used to neutralize the system. As elucidated in work by Blumberger and colleagues,^63^ (i) the large size of the simulated periodic box and (ii) the use of Na^+^ and Cl^–^ ions for neutralization make it such that these corrections to the redox properties are negligible (for further discussion on these corrections, see the Supporting Information).^63^

## Results and Discussion

### Structural Properties

The CURVES+ analysis of the classical simulations provides comparisons between wild-type and defect-packed DNA systems across 41 unique structural DNA properties.^89,90^ Our findings suggest that the 8oxoguanine defect does not yield any significant structural changes to packed DNA systems. Though slight structural differences between wild-type and defect systems are found for a few of the parameters explored, we observe that these differences are not significant enough for reliable exploitation by cellular repair processes. A full comparison of all 41 structural properties can be found in the Supporting Information. Here, we discuss a few key findings from our structural analysis.

**Figure 2:**
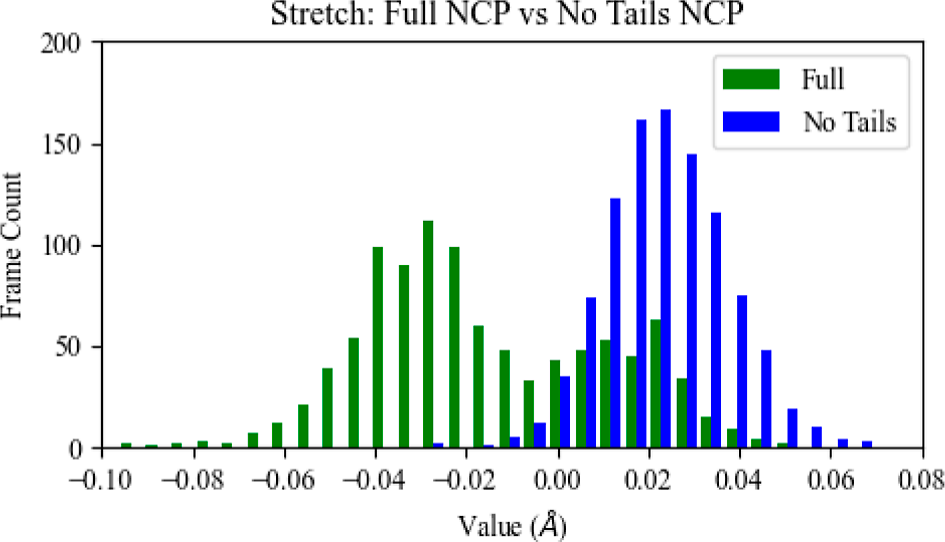
Population distributions for the DNA stretch structural property in the reduced wild-type system for the full 146 base pair NCP (full, green) and the same system omitting the first and final 20 base pairs (no tails, blue).

As can be anticipated, the tail regions of the NCP DNA have greater flexibility than the portions more closely interacting with the histone proteins. In macrostructures of chromatin, individual NCPs are linked by unraveled portions of DNA, which are expected to be more flexible than the DNA wrapped around the histone proteins, although likely less flexible than the cut version of the DNA tails modeled in our simulations. As our study is interested in the properties of packed DNA when oxidative damage occurs within the DNA strands wrapped around the histone proteins, we narrowed our global structural analysis to the structural properties of only 106 out of 146 base pairs of our NCP structure by eliminating from our analysis the structural property values of the first and last 20 base pairs that make up the flexible tail regions of our DNA systems. Figure 2 shows in a representative case how the omission of tail region base pairs yields a unimodal normal distribution of property values.

**Figure 3:**
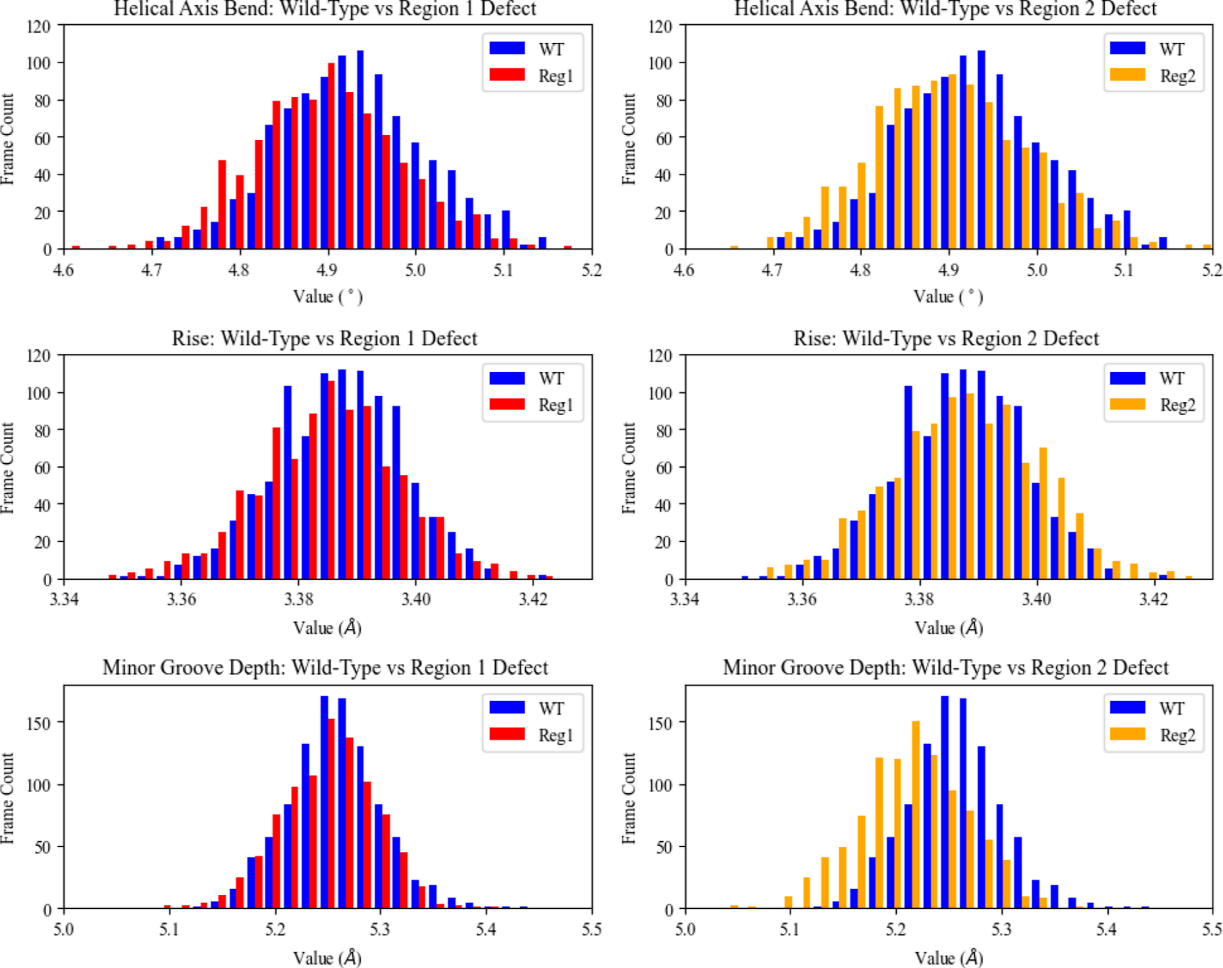
Population distributions of several structural properties (helical axis bend, rise, minor groove depth) for the wild-type system (WT, blue) vs the region 1 defect system (Reg1, red) and vs the region 2 defect system (Reg2, orange). The systems do not include in their analysis the 40 base pairs associated with tail regions. Associated means and standard deviations are as follows: helical axis bend wild-type 4.93 ± 0.082°; region 1 defect 4.89 ± 0.083°; region 2 defect 4.90 ± 0.088°; and rise wild-type 3.39 ± 0.011 Å; region 1 defect 3.38 ± 0.012 Å; region 2 defect 3.39 ± 0.013 Å; and minor groove depth wild-type 5.56 ± 0.044 Å; region 1 defect 5.25 ± 0.044 Å; region 2 defect 5.21 ± 0.050 Å.

**Figure 4:**
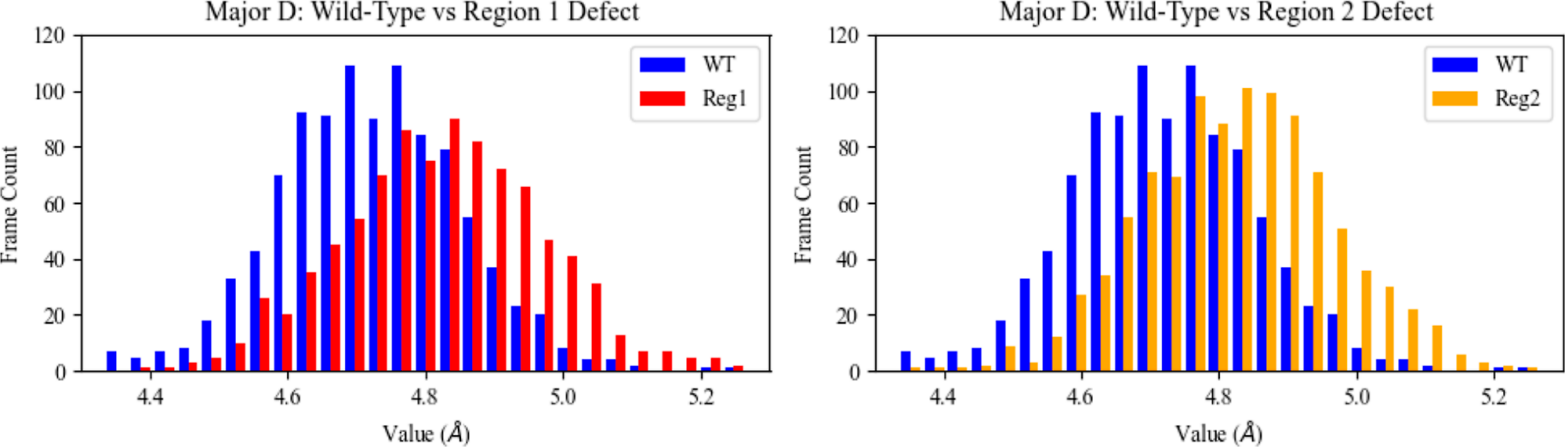
Population distributions of the major groove width and depth for the wild-type system (WT, blue) vs the region 1 defect system (Reg1, red) and for the wild-type system (WT, blue) vs the region 2 defect system (Reg2, orange). The systems do not include in their analysis the 40 base pairs associated with tail regions. Associated means and standard deviations are as follows: major groove depth wild-type 4.7 ± 0.13 Å; region 1 defect 4.8 ± 0.15 Å; region 2 defect 4.8 ± 0.14 Å.

Population distributions for each of the 41 structural property averages across their approximately 1000 frames were compared between the wild-type and two defect systems (see Supporting Information Figures S6–S23 with means and standard deviations in Supporting Information Table S8). For the majority of the properties, no differences were identified between the distributions of wild-type and defect systems. Figure 3 shows representative distributions exhibiting a high degree of overlap between systems. A difference in mean values that lies within the respective standard deviations is not considered significant, as a structural indicator would need to be present frequently enough with a unique enough value from the native structure to be reliably picked up by repair enzymes. Most of the parameters that do exhibit a difference in mean greater than one standard deviation occur in the region 2 systems, perhaps due to a greater flexibility in the defect base when solvent-exposed. However, Z-scores show little difference between these few distribution means (see Supporting Information Table S9). The only structural property distribution for which both defect systems simultaneously exhibited a slight but noticeable visual offset from the wild-type distribution was in the case of the major groove depth. However, observed differences in mean major groove values for all systems were less than the standard deviations, indicating the differences are not significantly and consistently noticeable. Furthermore, because regions 1 and 2 both reside in the minor groove, there is no obvious correlation between the location of regions 1 and 2 and a possible impact on the major groove. Figure 4 shows the slight offset for both defect systems in the major groove property.

To determine if differences in mean values which were larger than the respective standard deviations are affiliated with a specific local region of DNA, we also performed a side-by-side comparison of four 21 base pair sections of the NCP systems (see Supporting Information Figures S24–S33 with standard deviations and means in Supporting Information Table S10). The majority of differences in local distributions were localized in sections other than those containing the defect base itself. Perhaps a change in one location of the DNA could lead to changes 20–40 base pairs down the strand. However, for any structural effect to be a clear signal of damage, changes should systematically be observed near defects at any location of the NCP, which is not witnessed in this study.

Oxidative damage is the most common form of DNA damage, and yet, from our findings, the resulting damage products do not yield significant enough or location-independent structural changes at the global or local NCP level to serve as reliable detection indicators for all possible locations of oxidative damage within packed DNA. We now turn instead to the analysis of the redox properties of packed DNA as a potential feature to exploit for DNA damage detection.

### Redox Properties

#### Native Systems

**Figure 5:**
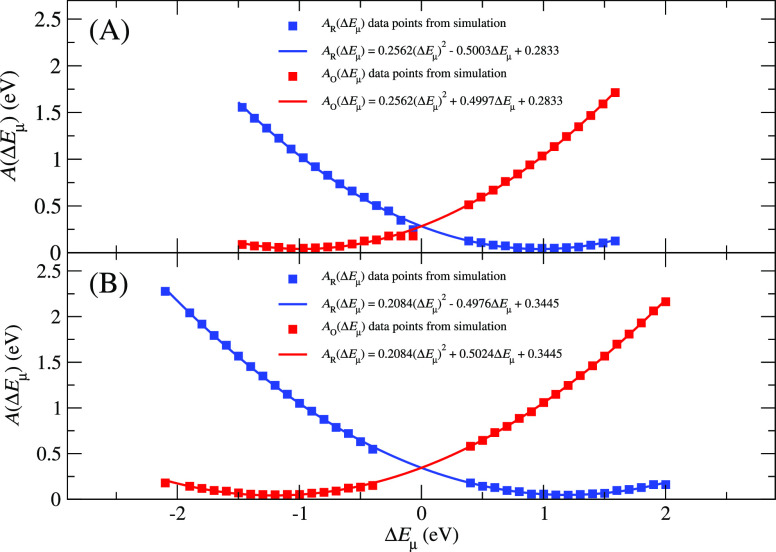
Free energy curves for the native NCP systems are shown for the G-rich regions 1 (A) and 2 (B). The solid lines are relationships derived from eq 4.

The time series of the VIEs and VEAs values used for the determination of the corresponding Δ*E* distributions and redox properties are available in the Supporting Information (Figure S1 in Supporting Information). For both cases (regions 1 and 2), the vertical energy gap distributions are Gaussian. They are available in Figure S3 of Supporting Information. For the first G-rich region (region 1), the average VIE and VEA, respectively, amount to 7.82 ± 0.24 and 5.81 ± 0.24 eV. Then, using eqs 1 and 2, Δ*A*_R→O_ = 6.81 ± 0.17 and λ = 1.01 ± 0.17 eV. For region 2, the average VIE and VEA are equal to 8.13 ± 0.24 and 5.77 ± 0.26 eV, and thus, Δ*A*_R→O_ = 6.95 ± 0.18 eV and λ = 1.18 ± 0.18 eV. The corresponding free energy curves for the reduced and oxidized states are shown in Figure 5 [insets (A,B) for regions 1 and 2, respectively].

#### Defect Systems

**Figure 6:**
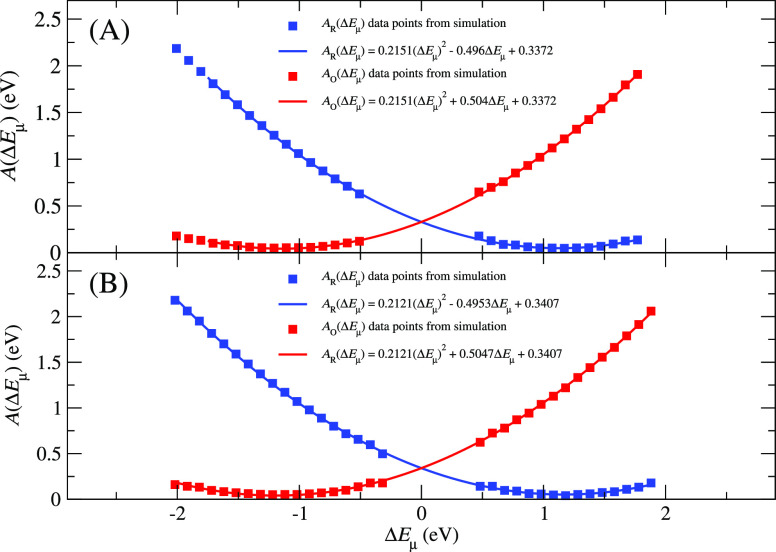
Free energy curves for the two defect NCP systems in which 8oxoG was placed in G-rich regions 1 (A) and 2 (B). The solid lines are relationships derived from eq 4.

The VIE and VEA time series and distributions for these systems are respectively depicted in Figures S2 and S4 of the Supporting Information. The Δ*E* distributions of both systems are Gaussian. For the first system (8oxoG defect in region 1), the average VIE is 6.88 ± 0.23 eV, while the average VEA amounts to 4.59 ± 0.27 eV. Therefore, Δ*A*_R→O_ = 5.73 ± 0.18 eV and λ = 1.14 ± 0.18 eV. For the second system (8oxoG in region 2), the average VIE and VEA, respectively, are 6.93 ± 0.23 and 4.60 ± 0.27 eV, and thus, Δ*A*_R→O_ = 5.77 ± 0.18 eV and λ = 1.17 ± 0.18 eV. The free energy curves for these two systems are shown in Figure 6 [insets (A,B)]. From the comparison of the calculated free energies of oxidation, we conclude that the presence of the 8oxoG defect impacts the redox properties more significantly than the location of the base in question.

### Assessment of the validity of Marcus Theory

As already mentioned above, in all four systems, the vertical energy gap distributions are Gaussian (Figures S3 and S4 of Supporting Information). Consequently, the free energy curves (Figures 5 and 6) are parabolic. Both of these outcomes are consistent with the validity of the Marcus theory. In addition, the very small differences seen in the respective VIE vs VEA standard deviations both in absolute values (0.24 eV vs 0.24 eV for native region 1, 0.24 eV vs 0.26 eV for native region 2, and 0.23 eV vs 0.27 eV for both 8oxoG-containing systems) and relative with respect to the average values (3.1% vs 4.1% for the native region 1, 3.0% vs 4.5% for the native region 2, and 3.3% vs 5.9% for both 8oxoG-containing systems) are also very well in line with the Marcus model.

Our systems’ adherence to Marcus theory is further supported by the fact that no significant rearrangements occur. Deviations from Marcus theory would be expected in cases where there is a major solvent rearrangement upon a change of oxidation states, for instance, due to the formation or breakage of a bond or a change in coordination number.^103^ The radial distribution functions of (i) the phosphorus atoms (P) of the DNA nucleotides belonging to the QM region and (ii) the water oxygen atoms (O_wat_), g(r P–O_wat_), were compared for the reduced and oxidized state of each of the four systems (native/8oxoG-containing for regions 1 and 2) and are shown in Supporting Information Figures S38 (region 1) and S39 (region 2). Analysis of these DNA-water radial distribution functions shows that the water reorganization upon change of the oxidation state is very small to negligible for all systems studied. The corresponding contributions to the computed Δ*A*_R→O_ and λ values are thus small. To take a closer look at solvent structure from the base-level, the average number of solvent oxygen atoms within 3.5 Å of the N7 atom of the central base (guanine or 8oxoguanine) was determined across the QM/MM MD trajectories (see Supporting Information Table S7). Local solvation changes very little between the reduced and oxidized states of the central base. This is compatible with the finding that the oxidation process of all systems studied follows Marcus theory and also validates the use of a nonpolarizable force field for water in this study.

Despite these confirmations, we employed the quadratic model developed by Matyushov and Voth^75,104^ in order to quantify the potentially missed “non-Marcus” Δ*A*_R→O_ contributions associated with differences in the solvent’s thermal fluctuations upon change of the solute’s oxidation state (due to, e.g., the use of a nonpolarizable force field for water). The calculation of this Δ*A*_R→O_ term^75,104^ (eq 7 in ref (26)) only requires the reorganization free energies of the reduced and oxidized states, which, as mentioned in the Theory Section, can be directly determined from the respective Δ*E* standard deviations. Employing this equation, this contribution to the redox free energy is equal to 0.01 and 0.04 eV for native regions 1 and 2, and 0.07 eV for both defect systems. Compared to the respective absolute values, these contributions are negligible, thus leading to final estimates of 6.82 ± 0.18 and 6.94 ± 0.21 eV for two native fragments and 5.73 ± 0.25 and 5.77 ± 0.25 eV for the two defect systems. This finding shows that for all systems studied, deviations from the Marcus assumption arising from altered solvent fluctuations due to different DNA and solvent polarization upon oxidation are negligible.

### Comparison of Reorganization Free Energies, Structural Interpretation, and Comparison with Bare DNA

As shown in the Redox Properties subsection, the reorganization free energy, λ, of the region 1 native and defect systems is 1.01 ± 0.17 eV and 1.14 ± 0.18 eV, respectively, while for region 2, λ amounts to 1.18 ± 0.17 eV for the native system and 1.17 ± 0.18 eV for the 8oxoG-containing system.

The relative absolute differences, 0.13 eV for region 1 and 0.01 eV for region 2, are even smaller than the ones reported for 8oxoG oxidation on bare DNA (0.25 eV).^26^ This is to be expected, given that in the NCP environment, DNA bases can interact with amino acids of the histone octamer, thus limiting their flexibility and reducing possible structural effects due to the change of the oxidation state. This is confirmed by a comparison of root-mean-square fluctuations (RMSFs) of the DNA nucleotides of the QM region in the reduced and oxidized states of each system (Tables S1 and S2 in the Supporting Information for regions 1 and 2, respectively) over the course of the QM/MM MD dynamics, which revealed small RMSFs and marginal differences between the two oxidation states.

Combined with the very small difference in water rearrangement upon oxidation of all systems studied (Figures S38–S39 and Table S7 of Supporting Information), this finding shows that the oxidation process of either G or 8oxoG leads to only a very small difference in structural reorganization. Furthermore, as previously discussed, differences in global structural properties between systems containing native guanine and systems containing defect 8oxoG are minimal. All together, this data suggests that changes in electrochemical properties provide a greater indicator of the presence of a defect than changes in structural properties.

An additional analysis was carried out on the QM/MM MD data in order to identify intermolecular DNA–protein interactions within the two G-rich QM regions. Multiple interactions were identified for region 1 systems (Table S3 of Supporting Information). Few DNA–protein interactions were identified with respect to the G-rich region 2, despite the fact that this region of DNA remained near the histone residues throughout the entirety of the simulation (Table S4 of Supporting Information). This finding indicates two G-rich regions of different structural exposure within the NCP, with region 1 being more buried and region 2 being more weakly coupled to protein residues in its vicinity and thus more exposed, respectively. This demonstrates that even local differences in intermolecular interactions within the NCP do not alter the large impact that 8oxoG has on the redox properties of NCP DNA, and thus, even more strongly, points to the recognition of 8oxoG in chromatin being possible via a DNA-mediated charge transfer (CT) scheme.

## Conclusions and Implications for 8oxoG Recognition in Chromatin

As discussed in the introduction, previous studies to assess whether defect-induced local differences in DNA redox chemistry can be exploited by BER enzymes for locating DNA lesions and initiating their repair process have so far been performed mostly on free, unpacked DNA. Among these studies, evidence indicates that a CT process could indeed be viable for the detection of oxidative DNA lesions.^20,26^ However, under cellular conditions, most of the genome is tightly packed within the NCP structure. This degree of compactness induces substantially different structural properties compared to free DNA. In combination with possible effects due to the interaction of DNA residues with amino acids from the histone octamer, the efficiency of a redox-based CT mechanism in chromatin might differ from the redox-based CT mechanism in free DNA. To our knowledge, the present investigation is the first computational study under solvated biological conditions with long thermal sampling in order to determine whether CT is a viable mechanism within an NCP structure.

To assess potential effects depending on the local environment, two distinct sites within the NCP were considered for 8oxoG insertion. In both cases, a highly pronounced relative difference in Δ*A*_R→O_ of approximately 1 eV has been found between the wild-type and defect-containing systems. On the other hand, the analysis of 41 DNA structural properties showed that their average values on a 1 μs time scale at 300 K differed very little between the wild-type and two defect systems. Further, the reorganization free energies, λ, differ only slightly between native and 8oxoG-containing NCPs (absolute relative changes of 0.13 and 0.01 eV, respectively). This is consistent with the small relative changes observed for solvent reorganization upon the oxidation of the native versus the defect systems (Figures S38–S39 in the Supporting Information). It is also in line with the very similar and small RMSF differences (reported in Tables S1 and S2 in Supporting Information) for DNA bases belonging to the respective QM regions. Moreover, with the analysis of DNA–protein contacts within the QM region of all NCP systems in consideration (Tables S3–S4 in the Supporting Information), it becomes clear that regardless of the exact position of the defect, the presence of the histone octamer has a minimal effect on the 8oxoG-induced changes of DNA redox properties in NCP. In addition, when compared to free, unpacked DNA,^26^ the smaller λ values reported herein demonstrate that the presence of the protein octamer further reduces possible relative structural differences upon oxidation.

The ensemble of these findings suggests that a CT-based detection of 8oxoG in chromatin is possible and relies on the differences in DNA redox chemistry between natural and defect-containing systems, regardless of the exact defect position within the NCP and the presence or absence of specific DNA–protein interactions. Furthermore, the lack of measurable structural changes at the NCP-level due to the 8oxoG oxidative lesion suggests that structural properties alone are not reliable indicators of DNA damage. The findings reported herein, highlighted by the relative differences of almost 1 eV in Δ*A*_R→O_, strongly indicate that detection of 8oxoG via a DNA-mediated hole transfer scheme, as proposed by Barton and co-workers, is also possible within the NCP, with the two BER enzymes likely being bound to different linker DNA fragments and scanning packed DNA between them. While the redox-based detection of oxidative defects seems unaffected in packed DNA, the overall repair process might be less efficient as the efficiency of 8oxoG removal is experimentally reported to decrease if it occurs in buried NCP regions.^40−43^ Still, our findings suggest that this decrease in efficiency should not be attributed to the detection scheme since Δ*A*_R→O_ decreases equally in both NCP regions considered here, and the detection of 8oxoG would thus be just as efficient. Instead, the decrease in 8oxoG removal efficiency is more likely due to the repair process because of the inaccessibility of buried NCP regions to binding by repair enzymes.

Experimental efforts to measure the redox potential of native vs 8oxoG-containing NCPs (with 8oxoG inserted in different NCPs and various exposed and buried sites within them) as well as the efficiency of hole transport (ideally with donors and acceptors that do not distort the NCP structure) would significantly contribute to furthering insight on the detection, recognition, and repair of 8oxoG by BER enzymes in the NCP structure. Ultimately, more investigations focused on various DNA lesions will provide a more comprehensive and generalized picture of how defect bases are detected and repaired in chromatin.

## Data Availability

Molecular dynamics (classical and QM/MM) and wave function optimization input parameter files, starting structures, analysis scripts, and some raw data are available in the GitHub and Zenodo repositories for this publication. Representative trajectory frames are available only in the Zenodo repository for this publication. It is available free of charge on GitHub https://github.com/lcbc-epfl/packed_dna_8oxog and on Zenodo https://doi.org/10.5281/zenodo.7705044 under CC BY 4.0.

## Supporting Information Available

Vertical energy gap distributions, RMSFs of DNA residues belonging in the QM region of all systems, identified DNA–protein close contacts, average hydrogen-bonding interactions with the defect regions and solvent molecules, average potential hydrogen-bonding partners within 0.35 nm of the central base of QM region (G or 8oxoG), number of average solvent oxygen atoms within 3.5 Å of the N7 atom of the central base, all 41 structural parameter distribution plots, select section-by-section structural parameter plots, all mean and standard deviations of structural parameter distributions, Z-scores for selected parameter distributions, RMSF plots of base flexibility for four DNA sections, and DNA-water radial distribution functions for the phosphorus atoms of the DNA backbone near the quantum region and oxygen atoms in water (PDF)
Molecular dynamics (classical and QM/MM) and wave function optimization input parameter files, starting structures, analysis scripts, and raw data (ZIP)

## References

[ref1] Cadet J.; Davies K. J. Oxidative DNA damage & repair: An introduction. Free Radic. Biol. Med. 2017, 107, 2–12. 10.1016/j.freeradbiomed.2017.03.030. 28363603 PMC5510741

[ref2] Cooke M. S.; Evans M. D.; Dizdaroglu M.; Lunec J. Oxidative DNA damage: mechanisms, mutation, and disease. FASEB J. 2003, 17, 1195–1214. 10.1096/fj.02-0752rev. 12832285

[ref3] Kryston T. B.; Georgiev A. B.; Pissis P.; Georgakilas A. G. Role of oxidative stress and DNA damage in human carcinogenesis. Mutat. Res., Fundam. Mol. Mech. Mutagen. 2011, 711, 193–201. 10.1016/j.mrfmmm.2010.12.016. 21216256

[ref4] Hush N. S.; Cheung A. S. Ionization potentials and donor properties of nucleic acid bases and related compounds. Chem. Phys. Lett. 1975, 34, 11–13. 10.1016/0009-2614(75)80190-4.

[ref5] Paukku Y.; Hill G. Theoretical determination of one-electron redox potentials for DNA bases, base Pairs, and stacks. J. Phys. Chem. A 2011, 115, 4804–4810. 10.1021/jp201281t. 21500846

[ref6] Pluhařová E.; Jungwirth P.; Bradforth S. E.; Slavíček P. Ionization of purine tautomers in nucleobases, nucleosides, and nucleotides: From the gas phase to the aqueous environment. J. Phys. Chem. B 2011, 115, 1294–1305. 10.1021/jp110388v. 21247073

[ref7] Seidel C. A.; Schulz A.; Sauer M. H. Nucleobase-specific quenching of fluorescent dyes. 1. Nucleobase one-electron redox potentials and their Correlation with static and dynamic quenching efficiencies. J. Phys. Chem. 1996, 100, 5541–5553. 10.1021/jp951507c.

[ref8] Fukuzumi S.; Miyao H.; Ohkubo K.; Suenobu T. Electron-transfer oxidation properties of DNA bases and DNA oligomers. J. Phys. Chem. A 2005, 109, 3285–3294. 10.1021/jp0459763. 16833661

[ref9] Heller A. Spiers Memorial Lecture. On the hypothesis of cathodic protection of genes. Faraday Discuss. 2000, 116, 1–13. 10.1039/b006196o. 11197472

[ref10] Beard W. A.; Batra V. K.; Wilson S. H. DNA polymerase structure-based insight on the mutagenic properties of 8-oxoguanine. Mutat. Res. - Genet. Toxicol. Environ. Mutagen. 2010, 703, 18–23. 10.1016/j.mrgentox.2010.07.013. PMC3023916 20696268

[ref11] van Loon B.; Markkanen E.; Hübscher U. Oxygen as a friend and enemy: How to combat the mutational potential of 8-oxo-guanine. DNA Repair 2010, 9, 604–616. 10.1016/j.dnarep.2010.03.004. 20399712

[ref12] Prat F.; Houk K. N.; Foote C. S. Effect of guanine stacking on the oxidation of 8-oxoguanine in B-DNA. J. Am. Chem. Soc. 1998, 120, 845–846. 10.1021/ja972331q.

[ref13] Pacheco-Ortín S.; Gaitán Lozano R.; Agacino Valdés E. Possible DNA damage by oxidation products of guanine: A density functional and electron propagator theoretical study. Int. J. Quantum Chem. 2012, 112, 2840–2847. 10.1002/qua.24001.

[ref14] Palivec V.; Pluhařová E.; Unger I.; Winter B.; Jungwirth P. DNA lesion can facilitate base ionization: Vertical ionization energies of aqueous 8-oxoguanine and its nucleoside and nucleotide. J. Phys. Chem. B 2014, 118, 13833–13837. 10.1021/jp5111086. 25390766

[ref15] Diamantis P.; Tavernelli I.; Rothlisberger U. Vertical Ionization Energies and Electron Affinities of Native and Damaged DNA Bases, Nucleotides, and Pairs from Density Functional Theory Calculations: Model Assessment and Implications for DNA Damage Recognition and Repair. J. Chem. Theory Comput. 2019, 15, 2042–2052. 10.1021/acs.jctc.8b00645. 30681847

[ref16] Sugiyama H.; Saito I. Theoretical studies of GG-specific photocleavage of DNA via electron transfer: Significant lowering of ionization potential and 5′-localization of HOMO of stacked GG bases in B-form DNA. J. Am. Chem. Soc. 1996, 118, 7063–7068. 10.1021/ja9609821.

[ref17] Steenken S.; Jovanovic S. V.; Bietti M.; Bernhard K. The Trap Depth (in DNA) of 8-Oxo-7,8-dihydro-2‘deoxyguanosine as Derived from Electron-Transfer Equilibria in Aqueous Solution. J. Am. Chem. Soc. 2000, 122, 2373–2374. 10.1021/ja993508e.

[ref18] Gasper S. M.; Schuster G. B. Intramolecular photoinduced electron transfer to anthraquinones linked to duplex DNA: The effect of gaps and traps on long-range radical cation migration. J. Am. Chem. Soc. 1997, 119, 12762–12771. 10.1021/ja972496z.

[ref19] Fujitsuka M.; Majima T. Hole and excess electron transfer dynamics in DNA. Phys. Chem. Chem. Phys. 2012, 14, 11234 10.1039/c2cp41576c. 22806184

[ref20] Boal A. K.; Barton J. K. Electrochemical detection of lesions in DNA. Bioconjugate Chem. 2005, 16, 312–321. 10.1021/bc0497362. 15769084

[ref21] Boal A. K.; Genereux J. C.; Sontz P. A.; Gralnick J. A.; Newman D. K.; Barton J. K. Redox signaling between DNA repair proteins for efficient lesion detection. Proc. Natl. Acad. Sci. U.S.A. 2009, 106, 15237–15242. 10.1073/pnas.0908059106. 19720997 PMC2741234

[ref22] Genereux J. C.; Boal A. K.; Barton J. K. DNA-mediated charge transport in redox sensing and signaling. J. Am. Chem. Soc. 2010, 132, 891–905. 10.1021/ja907669c. 20047321 PMC2902267

[ref23] Grodick M. A.; Segal H. M.; Zwang T. J.; Barton J. K. DNA-Mediated Signaling by Proteins with 4Fe-4S Clusters Is Necessary for Genomic Integrity. J. Am. Chem. Soc. 2014, 136, 6470–6478. 10.1021/ja501973c. 24738733 PMC4017601

[ref24] O’Brien E.; Silva R. M. B.; Barton J. K. Redox Signaling through DNA. Isr. J. Chem. 2016, 56, 705–723. 10.1002/ijch.201600022. 28090121 PMC5225960

[ref25] Boal A. K.; Genereux J. C.; Sontz P. A.; Gralnick J. A.; Newman D. K.; Barton J. K.; Boalaf A. K.; Généreux J. C.; Sontza P. A.; Gralnickb J. A.; Newman D. K.; Barton J. K. Redox signaling between DNA repair proteins for efficient lesion detection. Proc. Natl. Acad. Sci. 2009, 106, 15237–15242. 10.1073/pnas.0908059106. 19720997 PMC2741234

[ref26] Diamantis P.; Tavernelli I.; Rothlisberger U. Redox Properties of Native and Damaged DNA from Mixed Quantum Mechanical/Molecular Mechanics Molecular Dynamics Simulations. J. Chem. Theory Comput. 2020, 16, 6690–6701. 10.1021/acs.jctc.0c00568. 32926773

[ref27] Eriksen K. A. Location of DNA damage by charge exchanging repair enzymes: Effect of cooperativity on location time. Theor. Biol. Med. Model. 2005, 15, 2 10.1186/1742-4682-2-15. PMC1142343 15819980

[ref28] Lee M. H.; Brancolini G.; Gutierrez R.; Di Felice R.; Cuniberti G. Probing charge transport in oxidatively damaged DNA sequences under the influence of structural fluctuations. J. Phys. Chem. B 2012, 116, 10977–10985. 10.1021/jp2091544. 22679932

[ref29] Wohlgamuth C. H.; McWilliams M. A.; Slinker J. D. Temperature dependence of electrochemical DNA charge transport: Influence of a mismatch. Anal. Chem. 2013, 85, 1462–1467. 10.1021/ac302508f. 23252597

[ref30] Hickerson R. P.; Prat F.; Muller J. G.; Foote C. S.; Burrows C. J. Sequence and stacking dependence of 8-oxoguanine oxidation: Comparison of one-electron vs singlet oxygen mechanisms. J. Am. Chem. Soc. 1999, 121, 9423–9428. 10.1021/ja991929q.

[ref31] Shukla L. I.; Adhikary A.; Pazdro R.; Becker D.; Sevilla M. D. Formation of 8-oxo-7,8-dihydroguanine-radicals in γ-irradiated DNA by multiple one-electron oxidations. Nucleic Acids Res. 2004, 32, 6565–6574. 10.1093/nar/gkh989. 15601999 PMC545457

[ref32] Davey C.; Sargent D.; Luger K.; Maeder A.; Richmond T. Solvent mediated interactions in the structure of the nucleosome core particle at 1.9 a resolution. J. Mol. Biol. 2002, 319, 1097–1113. 10.1016/S0022-2836(02)00386-8. 12079350

[ref33] Widom J. Structure, dynamics, and function of chromatin in vitro. Annu. Rev. Biophys. Biomol. Struct. 1998, 27, 285–327. 10.1146/annurev.biophys.27.1.285. 9646870

[ref34] Wolffe A. Chromatin Structure and Function; San Diego Academic Press, Inc., 1992.

[ref35] Nunez M. E.; Noyes K. T.; Barton J. K. Oxidative charge transport through DNA in nucleosome core particles. Chem. Biol. 2002, 9, 403–415. 10.1016/S1074-5521(02)00121-7. 11983330

[ref36] Nakatani K.; Dohno C.; Ogawa A.; Saito I. Suppression of DNA-mediated charge transport by BamHI binding. Chem. Biol. 2002, 9, 361–366. 10.1016/S1074-5521(02)00119-9. 11927261

[ref37] Bjorklund C. C.; Davis W. B. Attenuation of DNA charge transport by compaction into a nucleosome core particle. Nucleic Acids Res. 2006, 34, 1836–1846. 10.1093/nar/gkl030. 16595797 PMC1428796

[ref38] Voityuk A. A.; Davis W. B. Hole transfer energetics in structurally distorted DNA: The nucleosome core particle. J. Phys. Chem. B 2007, 111, 2976–2985. 10.1021/jp066470i. 17388433

[ref39] Bjorklund C. C.; Davis W. B. Stable DNA - Protein Cross-Links Are Products of DNA Charge Transport in a Nucleosome Core Particle. Biochemistry 2007, 46, 10745–10755. 10.1021/bi700475b. 17760420

[ref40] Cook J. C.; Delaney S. The Domino Effect: Nucleosome Dynamics and the Regulation of Base Excision Repair Enzymes. DNA 2022, 2, 248–263. 10.3390/dna2040018.

[ref41] Amouroux R.; Campalans A.; Epe B.; Radicella J. P. Oxidative stress triggers the preferential assembly of base excision repair complexes on open chromatin regions. Nucleic Acids Res. 2010, 38, 2878–2890. 10.1093/nar/gkp1247. 20071746 PMC2875005

[ref42] Menoni H.; Shukla M. S.; Gerson V.; Dimitrov S.; Angelov D. Base excision repair of 8-oxoG in dinucleosomes. Nucleic Acids Res. 2012, 40, 692–700. 10.1093/nar/gkr761. 21930508 PMC3258150

[ref43] Davis W. B.; Bjorklund C. C.; Deline M. Probing the effects of DNA-protein interactions on DNA hole transport: the N-terminal histone tails modulate the distribution of oxidative damage and chemical lesions in the nucleosome core particle. Biochemistry 2012, 51, 3129–3142. 10.1021/bi201734c. 22409399

[ref44] Nilsen H.; Lindahl T.; Verreault A. DNA base excision repair of uracil residues in reconstituted nucleosome core particles. EMBO J. 2002, 21, 5943–5952. 10.1093/emboj/cdf581. 12411511 PMC131078

[ref45] Beard B. C.; Wilson S. H.; Smerdon M. J. Suppressed catalytic activity of base excision repair enzymes on rotationally positioned uracil in nucleosomes. Proc. Natl. Acad. Sci. U.S.A. 2003, 100, 7465–7470. 10.1073/pnas.1330328100. 12799467 PMC164609

[ref46] Hinz J. M.; Rodriguez Y.; Smerdon M. J. Rotational dynamics of DNA on the nucleosome surface markedly impact accessibility to a DNA repair enzyme. Proc. Natl. Acad. Sci. U.S.A. 2010, 107, 4646–4651. 10.1073/pnas.0914443107. 20176960 PMC2842065

[ref47] Cole H. A.; Tabor-Godwin J. M.; Hayes J. J. Uracil DNA glycosylase activity on nucleosomal DNA depends on rotational orientation of targets. J. Biol. Chem. 2010, 285, 2876–2885. 10.1074/jbc.M109.073544. 19933279 PMC2807341

[ref48] Rodriguez Y.; Smerdon M. J. The structural location of DNA lesions in nucleosome core particles determines accessibility by base excision repair enzymes. J. Biol. Chem. 2013, 288, 13863–13875. 10.1074/jbc.M112.441444. 23543741 PMC3650422

[ref49] Meas R.; Smerdon M. J. Nucleosomes determine their own patch size in base excision repair. Sci. Rep. 2016, 6, 27122 10.1038/srep27122. 27265863 PMC4893620

[ref50] Tiwari V.; Wilson D. M. I. DNA Damage and Associated DNA Repair Defects in Disease and Premature Aging. Am. J. Hum. Genet. 2019, 105, 237–257. 10.1016/j.ajhg.2019.06.005. 31374202 PMC6693886

[ref51] Norabuena E. M.; Barnes Williams S.; Klureza M. A.; Goehring L. J.; Gruessner B.; Radhakrishnan M. L.; Jamieson E. R.; Nunez M. E. Effect of the Spiroiminodihydantoin Lesion on Nucleosome Stability and Positioning. Biochemistry 2016, 55, 2411–2421. 10.1021/acs.biochem.6b00093. 27074396 PMC6299323

[ref52] Hazra T. K.; Das A.; Das S.; Choudhury S.; Kow Y. W.; Roy R. Oxidative DNA damage repair in mammalian cells: A new perspective. DNA Repair 2007, 6, 470–480. 10.1016/j.dnarep.2006.10.011. 17116430 PMC2702509

[ref53] Warshel A. Dynamics of reactions in polar solvents. Semiclassical trajectory studies of electron-transfer and proton-transfer reactions. J. Phys. Chem. 1982, 86, 2218–2224. 10.1021/j100209a016.

[ref54] King G.; Warshel A. Investigation of the free energy functions for electron transfer reactions. J. Chem. Phys. 1990, 93, 8682–8692. 10.1063/1.459255.

[ref55] Marcus R. A. On the Theory of Oxidation-Reduction Reactions Involving Electron Transfer. I. J. Chem. Phys. 1956, 24, 966–978. 10.1063/1.1742723.

[ref56] Marcus R. A. Electrostatic free energy and other properties of states having nonequilibrium polarization. I. J. Chem. Phys. 1956, 24, 979–989. 10.1063/1.1742724.

[ref57] Marcus R. A. On the theory of oxidation-reduction reactions involving electron transfer. III. applications to data on the rates of organic redox reactions. J. Chem. Phys. 1957, 26, 872–877. 10.1063/1.1743424.

[ref58] Marcus R. A. On the Theory of Electron-Transfer Reactions. VI. Unified Treatment for Homogeneous and Electrode Reactions. J. Chem. Phys. 1965, 43, 679–701. 10.1063/1.1696792.

[ref59] Blumberger J.; Tavernelli I.; Klein M. L.; Sprik M. Diabatic free energy curves and coordination fluctuations for the aqueous Ag^+^/Ag^2+^ redox couple: A biased Born-Oppenheimer molecular dynamics investigation. J. Chem. Phys. 2006, 124, 064507 10.1063/1.2162881. 16483220

[ref60] Cascella M.; Magistrato A.; Tavernelli I.; Carloni P.; Rothlisberger U. Role of protein frame and solvent for the redox properties of azurin from Pseudomonas aeruginosa. Proc. Natl. Acad. Sci. U.S.A. 2006, 103, 19641–19646. 10.1073/pnas.0607890103. 17179046 PMC1705813

[ref61] Sulpizi M.; Raugei S.; VandeVondele J.; Carloni P.; Sprik M. Calculation of redox properties: Understanding short- and long-range effects in rubredoxin. J. Phys. Chem. B 2007, 111, 3969–3976. 10.1021/jp067387y. 17388622

[ref62] Annapureddy H. V.; Margulis C. J. Controlling the outcome of electron transfer reactions in ionic liquids. J. Phys. Chem. B 2009, 113, 12005–12012. 10.1021/jp905144n. 19663446

[ref63] Seidel R.; Faubel M.; Winter B.; Blumberger J. Single–ion reorganization free energy of aqueous Ru(bpy)32+/3+and Ru(H_2_O)_6_^2+/3+^rom photoemission spectroscopy and density functional molecular dynamics simulation. J. Am. Chem. Soc. 2009, 131, 16127–16137. 10.1021/ja9047834. 19831354

[ref64] Willard A. P.; Reed S. K.; Madden P. A.; Chandler D. Water at an electrochemical interface-a simulation study. Faraday Discuss. 2009, 141, 423–441. 10.1039/B805544K. 19227369

[ref65] Kılıç M.; Ensing B. First and second one-electron reduction of lumiflavin in water-a first principles molecular dynamics study. J. Chem. Theory Comput. 2013, 9, 3889–3899. 10.1021/ct400088g. 26592384

[ref66] Diamantis P.; Gonthier J. F.; Tavernelli I.; Rothlisberger U. Study of the redox properties of singlet and triplet tris(2,2’-bipyridine)ruthenium(II) ([Ru(bpy)_3_]^2+^) in aqueous solution by full quantum and mixed quantum/classical molecular dynamics simulations. J. Phys. Chem. B 2014, 118, 3950–3959. 10.1021/jp412395x. 24611869

[ref67] Pounds M. A.; Salanne M.; Madden P. A. Molecular aspects of the Eu^3+^/Eu^2+^ redox reaction at the interface between a molten salt and a metallic electrode. Mol. Phys. 2015, 113, 2451–2462. 10.1080/00268976.2015.1046526.

[ref68] Firmino T.; Mangaud E.; Cailliez F.; Devolder A.; Mendive-Tapia D.; Gatti F.; Meier C.; Desouter-Lecomte M.; De La Lande A. Quantum effects in ultrafast electron transfers within cryptochromes. Phys. Chem. Chem. Phys. 2016, 18, 21442–21457. 10.1039/C6CP02809H. 27427185

[ref69] Chakraborty R.; Ghosh D. The effect of sequence on the ionization of guanine in DNA. Phys. Chem. Chem. Phys. 2016, 18, 6526–6533. 10.1039/C5CP07804K. 26864778

[ref70] Kılıç M.; Ensing B. Microscopic picture of the solvent reorganization during electron transfer to flavin in water. J. Phys. Chem. B 2019, 123, 9751–9761. 10.1021/acs.jpcb.9b07250. 31647869

[ref71] Luger K.; Mäder A. W.; Richmond R. K.; Sargent D. F.; Richmond T. J. Crystal structure of the nucleosome core particle at 2.8 Å resolution. Nature 1997, 389, 251–260. 10.1038/38444. 9305837

[ref72] Alberts B.; Johnson A.; Lewis J.; Raff M.; Roberts K.; Walter P. Membrane Transport of Small Molecules and the Electrical Properties of Small Membranes. Molecular Biology of the Cell, 4th ed.; Garland Science, New York, 2002; pp 615–657.

[ref73] Saito I.; Nakamura T.; Nakatani K.; Yoshioka Y.; Yamaguchi K.; Sugiyama H. Mapping of the Hot Spots for DNA Damage by One-Electron Oxidation: Efficacy of GG Doublets and GGG Triplets as a Trap in Long-Range Hole Migration. J. Am. Chem. Soc. 1998, 120, 12686–12687. 10.1021/ja981888i.

[ref74] Li X.; Peng Y.; Ren J.; Qu X. Effect of DNA flanking sequence on charge transport in short DNA duplexes. Biochemistry 2006, 45, 13543–13550. 10.1021/bi061103i. 17087508

[ref75] Small D. W.; Matyushov D. V.; Voth G. A. The theory of electron transfer reactions: What may be missing?. J. Am. Chem. Soc. 2003, 125, 7470–7478. 10.1021/ja029595j. 12797822

[ref76] Abraham M.; van der Spoel D.; Lindahl E.; Hess B.; The GROMACS Development Team. GROMACS User Manual. version 2019.4, 2019. http://www.gromacs.org.

[ref77] Van Der Spoel D.; Lindahl E.; Hess B.; Groenhof G.; Mark A.; Berendsen H. GROMACS: fast, flexible, and free. J. Comput. Chem. 2005, 26, 1701–1718. 10.1002/jcc.20291. 16211538

[ref78] Maier J. A.; Martinez C.; Kasavajhala K.; Wickstrom L.; Hauser K. E.; Simmerling C. ff14SB: Improving the Accuracy of Protein Side Chain and Backbone Parameters from ff99SB. J. Chem. Theory Comput. 2015, 11, 3696–3713. 10.1021/acs.jctc.5b00255. 26574453 PMC4821407

[ref79] Ivani I.; Dans P. D.; Noy A.; Perez A.; Faustino I.; Hospital A.; Walther J.; Andrio P.; Goni R.; Balaceanu A.; Portella G.; Battistini F.; Gelpi J. L.; Gonzalez C.; Vendruscolo M.; Laughton C. A.; Harris S. A.; Case D. A.; Orozco M. Parmbsc1: A refined force field for DNA simulations. Nat. Methods 2016, 13, 55–58. 10.1038/nmeth.3658. 26569599 PMC4700514

[ref80] Dans P. D.; Ivani I.; Hospital A.; Portella G.; Gonzalez C.; Orozco M. How accurate are accurate force-fields for B-DNA?. Nucleic Acids Res. 2017, 45, 4217–4230. 10.1093/nar/gkw1355. 28088759 PMC5397185

[ref81] Miller J. H.; Fan-Chiang C. C. P.; Straatsma T. P.; Kennedy M. A. 8-Oxoguanine enhances bending of DNA that favors binding to glycosylases. J. Am. Chem. Soc. 2003, 125, 6331–6336. 10.1021/ja029312n. 12785867

[ref82] Jorgensen W. L.; Chandrasekhar J.; Madura J. D.; Impey R. W.; Klein M. L. Comparison of simple potential functions for simulating liquid water. J. Chem. Phys. 1983, 79, 926–935. 10.1063/1.445869.

[ref83] Joung I. S.; Cheatham T. E. Determination of alkali and halide monovalent ion parameters for use in explicitly solvated biomolecular simulations. J. Phys. Chem. B 2008, 112, 9020–9041. 10.1021/jp8001614. 18593145 PMC2652252

[ref84] Hess B.; Bekker H.; Berendsen H. J. C.; Fraaije J. G. E. M. LINCS: A linear constraint solver for molecular simulations. J. Comput. Chem. 1997, 18, 1463–1472. 10.1002/(SICI)1096-987X(199709)18:12<1463::AID-JCC4>3.0.CO;2-H.

[ref85] Darden T.; York D.; Pedersen L. Particle mesh Ewald: An N.log(N) method for Ewald sums in large systems. J. Chem. Phys. 1993, 98, 10089–10092. 10.1063/1.464397.

[ref86] Hoover W. G. Canonical dynamics: Equilibrium phase-space distributions. Phys. Rev. A 1985, 31, 1695–1697. 10.1103/PhysRevA.31.1695. 9895674

[ref87] Parrinello M.; Rahman A. Crystal Structure and Pair Potentials: A Molecular-Dynamics Study. Phys. Rev. Lett. 1980, 45, 1196–1199. 10.1103/PhysRevLett.45.1196.

[ref88] Parrinello M.; Rahman A. Polymorphic transitions in single crystals: A new molecular dynamics method. J. Appl. Phys. 1981, 52, 7182–7190. 10.1063/1.328693.

[ref89] Lavery R.; Moakher M.; Maddocks J. H.; Petkeviciute D.; Zakrzewska K. Conformational analysis of nucleic acids revisited: Curves+. Nucleic Acids Res. 2009, 37, 5917–5929. 10.1093/nar/gkp608. 19625494 PMC2761274

[ref90] Blanchet C.; Pasi M.; Zakrzewska K.; Lavery R. CURVES+ web server for analyzing and visualizing the helical, backbone and groove parameters of nucleic acid structures. Nucleic Acids Res. 2011, 39, W68–W73. 10.1093/nar/gkr316. 21558323 PMC3125750

[ref91] The CPMD Consortium Page. The CPMD program is 2000–2019 jointly by IBM Corp. and by Max Planck Institute, Stuttgart. It is distributed free of charge to non-profit Organizations under the CPMD free licence, 2019. http://www.cpmd.org (accessed 2021-09-01).

[ref92] van Gunsteren W. F.; Berendsen H. J. C. Molecular Simulation (GROMOS) Library Manual; Biomos The Netherlands, Groningen, 1987; pp 1–221.

[ref93] van Gunsteren W. F.; Billeter S. R.; Eising A. A.; Hünenberger P. H.; Krüger P.; Mark A. E.; Scott W. R. P.; Tironi I. G. Biomolecular Simulation: The GROMOS96 Manual and User Guide; Vdf Hochschulverlag AG an der ETH Zürich: Zürich, Switzerland, 1996; pp 1–1042.

[ref94] Laio A.; VandeVondele J.; Rothlisberger U. A Hamiltonian electrostatic coupling scheme for hybrid Car-Parrinello molecular dynamics simulations. J. Chem. Phys. 2002, 116, 6941–6947. 10.1063/1.1462041.

[ref95] Colombo M. C.; Zumstein M.; VandeVondele J.; Sulpizi M.; Spiegel K.; Röhrig U.; Piana S.; Maurer P.; Magistrato A.; Laio A.; Guidoni L.; Röthlisberger U. Hybrid QM/MM Car-Parrinello simulations of catalytic and enzymatic reactions. Chimia 2002, 56, 13–19. 10.2533/000942902777680865.

[ref96] Brunk E.; Rothlisberger U. Mixed Quantum Mechanical/Molecular Mechanical Molecular Dynamics Simulations of Biological Systems in Ground and Electronically Excited States. Chem. Rev. 2015, 115, 6217–6263. 10.1021/cr500628b. 25880693

[ref97] Becke A. D. Density-Functional Exchange-Energy Approximation with Correct Asymptotic Behavior. Phys. Rev. A: At., Mol., Opt. Phys. 1988, 38, 3098–3100. 10.1103/PhysRevA.38.3098. 9900728

[ref98] Lee C.; Yang W.; Parr R. G. Development of the Colle- Salvetti Correlation-Energy Formula into a Functional of the Electron Density. Phys. Rev. B: Condens. Matter Mater. Phys. 1988, 37, 785–789. 10.1103/PhysRevB.37.785. 9944570

[ref99] von Lilienfeld O. A.; Tavernelli I.; Rothlisberger U.; Sebastiani D. Optimization of Effective Atom Centered Potentials for London Dispersion Forces in Density Functional Theory. Phys. Rev. Lett. 2004, 93, 153004 10.1103/PhysRevLett.93.153004. 15524874

[ref100] Lin I.-C.; Coutinho-Neto M. D.; Felsenheimer C.; von Lilienfeld O. A.; Tavernelli I.; Rothlisberger U. Library of dispersion-corrected atom-centered potentials for generalized gradient approximation functionals: Elements H, C, N, O, He, Ne, Ar, and Kr. Phys. Rev. B 2007, 75, 205131 10.1103/PhysRevB.75.205131.

[ref101] Troullier N.; Martins J. L. Efficient pseudopotentials for plane-wave calculations. Phys. Rev. B 1991, 43, 1993–2006. 10.1103/PhysRevB.43.1993. 9997467

[ref102] Car R.; Parrinello M. Unified Approach for Molecular Dynamics and Density-Functional Theory. Phys. Rev. Lett. 1985, 55, 2471–2474. 10.1103/PhysRevLett.55.2471. 10032153

[ref103] Vuilleumier R.; Tay K. A.; Jeanmairet G.; Borgis D.; Boutin A. Extension of Marcus picture for electron transfer reactions with large solvation changes. J. Am. Chem. Soc. 2012, 134, 2067–2074. 10.1021/ja2069104. 22148250

[ref104] Matyushov D. V.; Voth G. A. Modeling the free energy surfaces of electron transfer in condensed phases. J. Chem. Phys. 2000, 113, 5413–5424. 10.1063/1.1289886.

