# Supplementary material for: Redox-Based Defect Detection in Packed DNA: Insights from Hybrid Quantum Mechanical/Molecular Mechanics Molecular Dynamics Simulations
Source: J Chem Theory Comput. 2023 Nov 14;19(22):8434–45. doi: 10.1021/acs.jctc.3c01013 (PMC10687876; doi:10.1021/acs.jctc.3c01013)
Supplement: Supplementary file 1 — ct3c01013_si_001.pdf [file ct3c01013_si_001.pdf]

Supplementary Information
Redox-Based Defect Detection in Packed DNA: Insights from Hybrid Quantum Mechanical/Molecular Mechanics Molecular Dynamics Simulations

Murat Kılıç†, Polydefkis Diamantis†, Sophia K. Johnson, Oliver Toth, and Ursula Rothlisberger*

Laboratory of Computational Chemistry and Biochemistry, Institute of Chemical Sciences and Engineering, École Polytechnique Fédérale de Lausanne (EPFL), CH-1015 Lausanne, Switzerland

E-mail: ursula.roethlisberger@epfl.ch

Discussion on accounting for the finite size of the periodic simulation box:

In the literature, a set of corrections concerning the effects of a finite simulation box on redox calculations has been outlined (reference 63). First, the calculated reorganization energy lacks contributions from higher order solvation shell changes due to finite volume of the simulation box. To reduce errors in the reorganization energy, corrections must be explicitly added and/or the simulated system must be solvated well enough to mimic an "infinitely diluted" system. The current study utilizes a large simulation box (dimensions of 148 Å × 159 Å × 107 Å). Additionally, the system is thoroughly solvated with

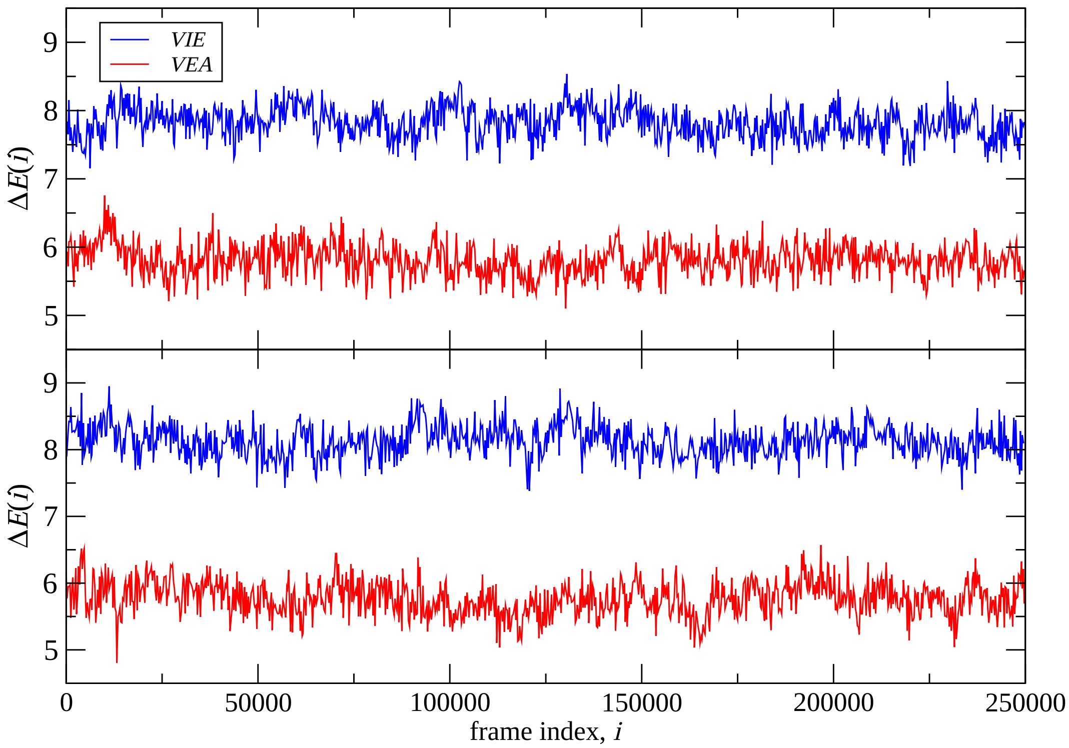

Figure S1: Time series of the vertical ionization energies (*VIE*s) and vertical electron affinities (*VEA*s) used for the determination of the vertical energy gap distributions and redox properties of the native G-rich **regions 1** (**top**) and **2** (**bottom**).

approximately 76500 water molecules. Together, this simulation box mimics an "infinitely diluted" system which mitigates the need for explicitly added corrections.

Second, charge neutrality must be enforced for the accurate calculation of redox properties. Without guaranteed charge neutrality, a correction term must be included when calculating reorganization energy values. The charge neutrality of the current study's simulation box is maintained through the explicit inclusion of sodium and chloride counterions which maintain an overall system charge of zero. Therefore, a correction term to account for differences across systems when calculating redox properties in this study is not necessary.

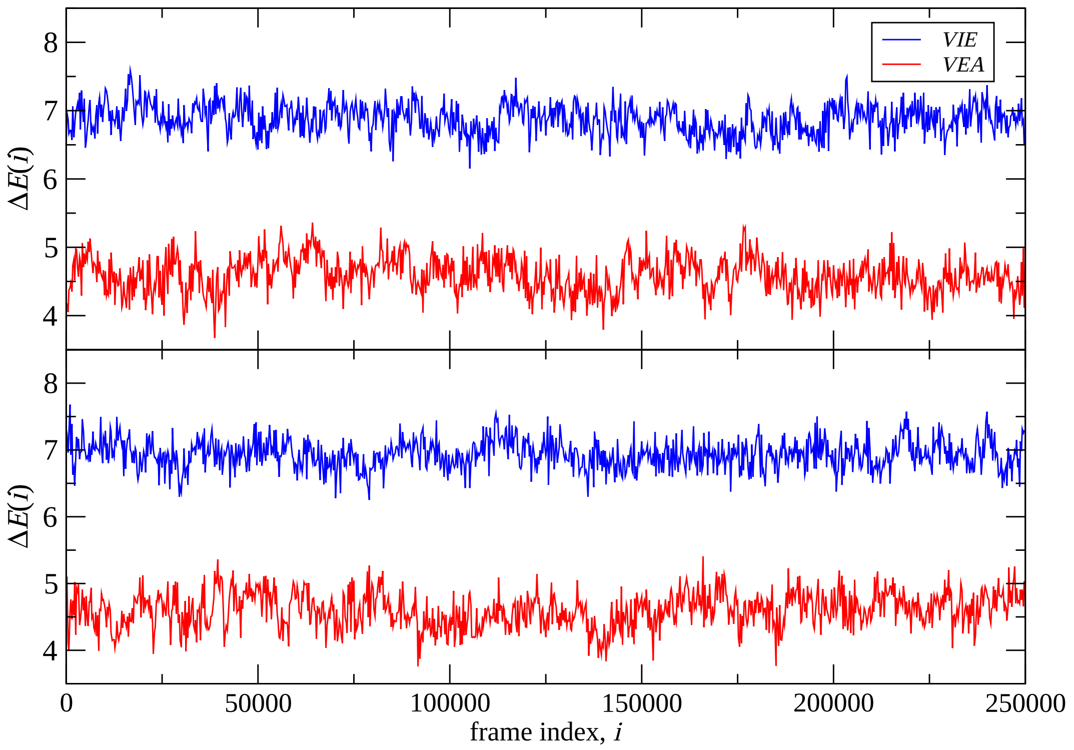

Figure S2: Time series of the *VIE*s and *VEA*s used for the determination of the vertical energy gap distributions and redox properties of the defect systems in which the 8-oxoguanine (8oxoG) base was placed in G-rich **regions 1** (**top**) and **2** (**bottom**).

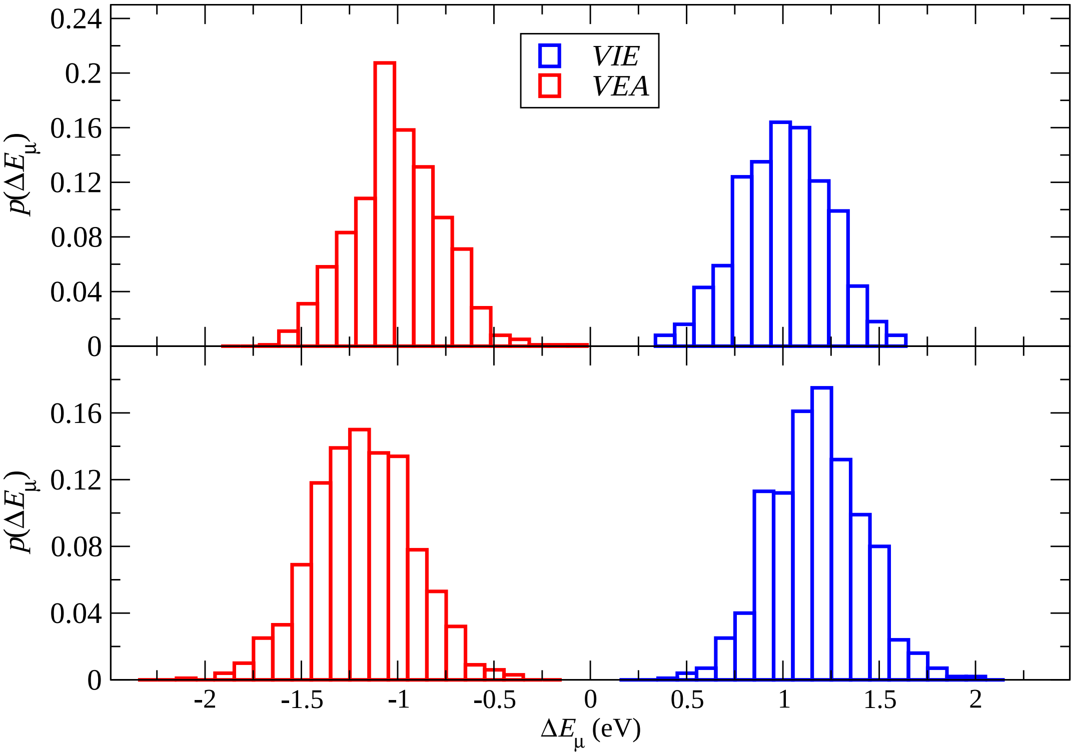

Figure S3: *VIE* and *VEA* distributions for the native G-rich **regions 1** (**top**) and **2** (**bottom**).

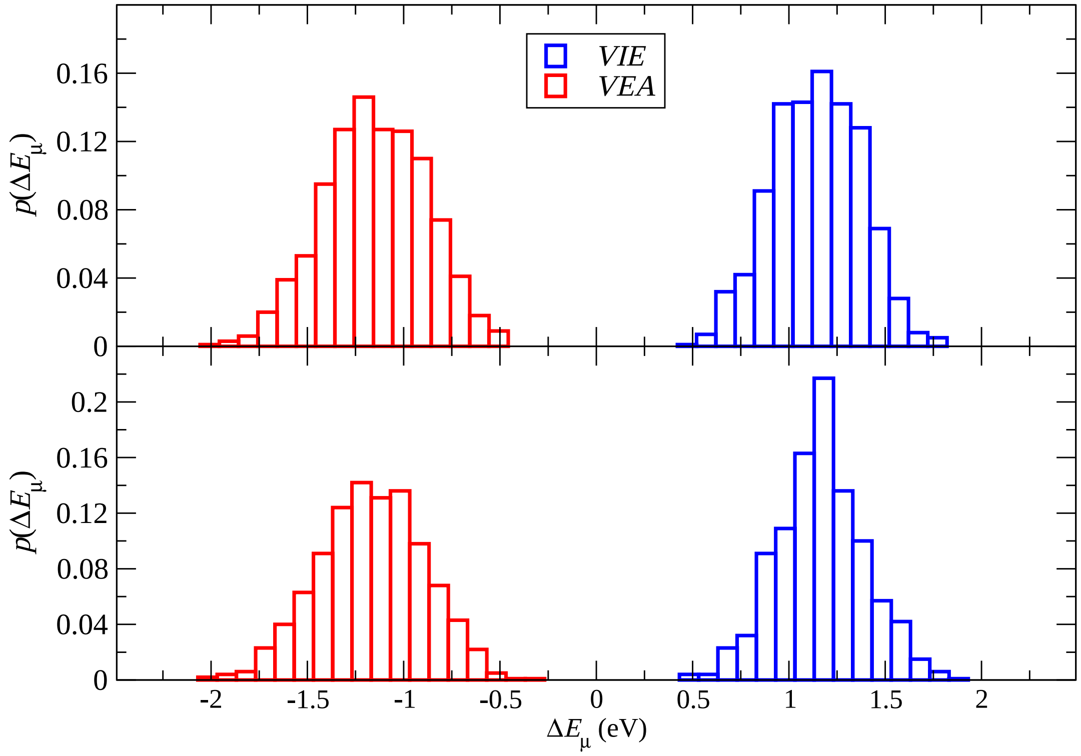

Figure S4: *VIE* and *VEA* distributions for the defect systems in which 8oxoG was placed in in G-rich **regions 1** (**top**) and **2** (**bottom**).

5' - ATCAATATCCACCTGCAGATTCTACCAAAAGTGTATTTGGAAACTGCTCCATCAAAAGG*CATGTTCAGCTGAA
TTCAGCTGAACATGCCTTTTGATGGAGCAGTTTCCAAATACACTTTTGGTAGAATCTGCAGGTGGATATTGAT - 3'

Figure S5: DNA sequence of strand 1, with the guanine marked at base 59 corresponding to the central base of **region 1**. Strand 2 has the same sequence in the 3' to 5' direction as strand 1 in the 5' to 3' direction. On strand 2 **region 2** has a central base at a reciprocal location to **region 1**.

Table S1: G-rich **region 1** systems: Root mean square fluctuations (RMSFs) for the DNA residues belonging in the QM region of the native (left) and 8oxoG-containing (right) systems.

Native				8oxoG			
Reduced		Oxidized		Reduced		Oxidized	
ResID	RMSF (Å)	ResID	RMSF (Å)	ResID	RMSF (Å)	ResID	RMSF (Å)
58	0.46	58	0.47	58	0.43	58	0.44
59	0.44	59	0.40	59	0.46	59	0.39
60	0.43	60	0.40	60	0.48	60	0.41
233	0.58	233	0.45	233	0.47	233	0.43
234	0.55	234	0.40	234	0.50	234	0.61
235	0.51	235	0.61	235	0.63	235	0.54

Table S2: G-rich **region 2** systems: Root mean square fluctuations (RMSFs) for the DNA residues belonging in the QM region of the native (left) and 8oxoG-containing (right) systems.

Native				8oxoG			
Reduced		Oxidized		Reduced		Oxidized	
ResID	RMSF (Å)	ResID	RMSF (Å)	ResID	RMSF (Å)	ResID	RMSF (Å)
87	0.52	87	0.58	87	0.46	87	0.49
88	0.48	88	0.48	88	0.42	88	0.40
89	0.53	89	0.48	89	0.44	89	0.39
204	0.67	204	0.45	204	0.42	204	0.50
205	0.44	205	0.45	205	0.38	205	0.44
206	0.56	206	0.44	206	0.43	206	0.48

Table S3: G-rich **region 1** systems: QM-treated DNA and neighboring protein residues sharing a strong intermolecular interaction in the native (left) and 8oxoG-containing (right) systems.

Native				8oxoG			
Reduced		Oxidized		Reduced		Oxidized	
ResID_{DNA}	$\mathrm{ResID}_{Pro.}$	ResID_{DNA}	$\mathrm{ResID}_{Pro.}$	ResID_{DNA}	$\mathrm{ResID}_{Pro.}$	ResID_{DNA}	$\mathrm{ResID}_{Pro.}$
58	–	58	–	58	–	58	–
59	–	59	–	59	–	59	318
60	407	60	401 / 407	60	318 / 401	60	318 / 407
233	–	233	–	233	–	233	–
234	–	234	–	234	–	234	–
235	391	235	–	235	–	235	–

Table S4: G-rich **region 2** systems: QM-treated DNA and neighboring protein residues sharing an intermolecular interaction in the native (left) and 8oxoG-containing (right) systems. With the exception of a strong (206-731) and a weak (204-688) interaction found for for the oxidized native system, no intermolecular interactions were identified.

Native				8oxoG			
Reduced		Oxidized		Reduced		Oxidized	
$ResID_{DNA}$	$ResID_{Pro.}$	$ResID_{DNA}$	$ResID_{Pro.}$	$ResID_{DNA}$	$ResID_{Pro.}$	$ResID_{DNA}$	$ResID_{Pro.}$
87	–	87	–	87	–	87	–
88	–	88	–	88	–	88	–
89	–	89	–	89	–	89	–
204	–	204	688	204	–	204	–
205	–	205	–	205	–	205	–
206	–	206	731	206	–	206	–

Table S5: The 3 DNA base pairs which comprise the G-rich **region 2** systems have a greater number of average H-bonding interactions with solvent molecules throughout the classical molecular dynamics simulation than the 3 DNA base pairs of the G-rich **region 1** systems indicating that **region 2** is more solvent-exposed than **region 1** regardless of the presence or absence of the 8oxoguanine defect. Average H-bonding interactions between the DNA bases and solvent molecules were calculated with GROMACS hydrogen bonding tool which counts viable hydrogen bonding interactions throughout a trajectory for selected donor and acceptor groups.

Native		8oxoG	
Region 1	Region 2	Region 1	Region 2
46.70	53.65	50.87	54.38

Table S6: The central guanine or 8oxoguanine which comprises the G-rich **region 2** systems have a greater number of average H-bonding eligible solvent partners within 0.35nm throughout the classical molecular dynamics simulation than the central guanine or 8oxoguanine of the G-rich **region 1** systems indicating that **region 2** is more solvent-exposed than **region 1** even on the central base level. Average H-bonding eligible partners between the DNA base and solvent molecules were calculated with GROMACS hydrogen bonding tool with counts viable hydrogen bonding partners within 0.35nm throughout a trajectory for selected donor and acceptor groups.

Native		8oxoG	
Region 1	Region 2	Region 1	Region 2
6.93	10.20	9.66	11.36

Table S7: Average number of oxygen solvent atoms within 3.5 Å of the N7 atom on the central base of the quantum region (guanine or 8oxoguanine) throughout the entire QM/MM MD trajectory. Local changes of the reduced versus oxidized base in terms of nearby solvent coordination is very low.

Native				8oxoG			
Region 1		Region 2		Region 1		Region 2	
Red	Ox	Red	Ox	Red	Ox	Red	Ox
1.6 ± 0.75	1.9 ± 0.65	2.1 ± 0.66	2.0 ± 0.73	2.0 ± 0.79	2.1 ± 0.83	2.1 ± 0.81	2.1 ± 0.78

Table S8: Means and standard deviations of DNA structural parameters.

	Parameter	Wild-Type	Region 1 Defect	Region 2 Defect
Intrabase Translational (Å)	Shear	-0.017 ± 0.0375	-0.0049 ± 0.03335	-0.0045 ± 0.03251
	Stretch	0.024 ± 0.0133	0.026 ± 0.0137	0.032 ± 0.0143
	Stagger	0.062 ± 0.0491	0.045 ± 0.0486	0.028 ± 0.0494
Intrabase Rotational (°)	Buckle	-0.25 ± 1.181	-0.92 ± 1.186	-0.48 ± 1.215
	Propeller	-9.4 ± 0.86	-9.1 ± 0.81	-9.5 ± 0.78
	Opening	1.5 ± 0.50	1.3 ± 0.53	1.2 ± 0.51
Interbase Translational (Å)	Shift	-0.0089 ± 0.03612	0.0051 ± 0.04081	-0.0073 ± 0.03876
	Slide	-0.25 ± 0.048	-0.25 ± 0.048	-0.25 ± 0.053
	Rise	3.39 ± 0.011	3.38 ± 0.012	3.39 ± 0.013
Interbase Rotational (°)	Tilt	0.014 ± 0.2473	0.031 ± 0.2664	0.033 ± 0.2608
	Roll	-0.27 ± 0.354	-0.26 ± 0.400	-0.055 ± 0.3771
	Twist	34.5 ± 0.10	34.4 ± 0.13	34.6 ± 0.12
Base-Axis Translational (Å)	XDisp	-0.41 ± 0.059	-0.44 ± 0.064	-0.48 ± 0.066
	YDisp	0.014 ± 0.0456	-0.0062 ± 0.05056	0.015 ± 0.0499
Base-Axis Rotational (°)	Inclination	-0.22 ± 0.577	-0.12 ± 0.656	0.23 ± 0.618
	Tip	-0.063 ± 0.4044	-0.057 ± 0.442	-0.12 ± 0.430
Strand 1 Torsional Angles (°)	Alpha1	-70. ± 2.3	-69 ± 2.4	-70. ± 2.0
	Beta1	76 ± 12.9	74 ± 12.4	80 ± 12.8
	Gamma1	53 ± 3.7	51 ± 4.6	55 ± 2.5
	Delta1	136 ± 1.2	136 ± 1.4	133 ± 1.3
	Epsilon1	-92 ± 11.8	-96 ± 11.4	-86 ± 11.9
	Zeta1	-53 ± 6.4	-55 ± 7.3	-56 ± 6.7
	Chi1	-113 ± 1.5	-113 ± 1.7	-113 ± 1.6
Strand 2 Torsional Angles (°)	Alpha2	-72 ± 2.1	-70. ± 2.2	-71 ± 2.1
	Beta2	80. ± 12.5	78 ± 13.3	77 ± 12.7
	Gamma2	51 ± 4.0	52 ± 3.6	53 ± 3.2
	Delta2	136 ± 1.2	136 ± 1.3	134 ± 1.3
	Epsilon2	-96 ± 11.5	-92 ± 12.2	-88 ± 11.8
	Zeta2	-51 ± 6.7	-55 ± 7.4	-56 ± 6.7
	Chi2	-112 ± 1.5	-112 ± 1.4	-112 ± 1.5
Strand 1 Sugar Pucker (°)	Phase1	117 ± 8.2	120. ± 8.2	117 ± 8.3
	Amplitude1	41.1 ± 0.49	41.2 ± 0.50	41.0 ± 0.51
Strand 2 Sugar Pucker (°)	Phase2	117 ± 8.7	116 ± 8.2	118 ± 8.3
	Amplitude2	41.4 ± 0.19	41.2 ± 0.53	41.2 ± 0.51
Helical Axis (Å; °)	H-Rise	3.36 ± 0.017	3.37 ± 0.020	3.34 ± 0.021
	H-Twist	35.30 ± 0.093	35.3 ± 0.12	35.5 ± 0.11
	Ax-Bend	4.93 ± 0.082	4.89 ± 0.083	4.90 ± 0.088
Minor Groove (Å)	Minor W	5.1 ± 0.12	5.1 ± 0.15	5.3 ± 0.12
	Minor D	5.26 ± 0.044	5.25 ± 0.044	5.21 ± 0.050
Major Groove (Å)	Major W	11.5 ± 0.13	11.6 ± 0.15	11.5 ± 0.14
	Major D	4.7 ± 0.13	4.8 ± 0.15	4.8 ± 0.14

Table S9: Z-scores for selected parameters whose difference in mean falls outside one or both of the individual distribution standard deviations.

Parameter	Z-Score
Twist Reg2	0.64
XDisp Reg2	0.79
Inclin. Reg2	0.53
Delta1 Reg2	1.7
Delta2 Reg2	1.1
H-Twi Reg2	1.4
Min.W Reg2	1.2
Maj.W R1	0.50
Maj.D R1	0.50
Maj.D R2	0.52

Table S10: Means and standard deviations of selected DNA structural parameters by section. Selected parameters exhibit differences in wild-type and defect mean greater than the associated distribution widths (standard deviations).

Parameter	Section 1	Section 2	Section 3	Section 4
Twist (°)				
WT	35.3 ± 0.42	34.7 ± 0.44	34.3 ± 0.35	34.8 ± 0.36
R2 Defect	35.8 ± 0.44	34.7 ± 0.42	34.6 ± 0.34	35.1 ± 0.41
XDisp (Å)				
WT	-0.19 ± 0.145	-0.21 ± 0.142	-0.39 ± 0.137	-0.283 ± 0.145
R2 Defect	-0.059 ± 0.1431	-0.44 ± 0.145	-0.41 ± 0.152	-0.27 ± 0.158
Inclin. (°)				
WT	-1.4 ± 1.35	-3.0 ± 1.36	-0.53 ± 1.522	-1.7 ± 1.35
R2 Defect	-0.83 ± 1.353	-3.0 ± 1.45	0.67 ± 1.334	-1.2 ± 1.29
Delta1 (°)				
WT	136 ± 2.6	137 ± 2.8	134 ± 3.1	137 ± 2.8
R2 Defect	136 ± 2.7	134 ± 3.4	132 ± 3.4	135 ± 3.0
Delta2 (°)				
WT	137 ± 2.7	138 ± 2.5	136 ± 3.2	137 ± 2.7
R2 Defect	137 ± 3.1	134 ± 2.8	133 ± 3.4	135 ± 3.1
H-Twi (°)				
WT	35.9 ± 0.40	35.7 ± 0.40	35.2 ± 0.33	35.5 ± 0.35
R2 Defect	36.4 ± 0.39	35.7 ± 0.38	35.5 ± 0.33	35.7 ± 0.39
Min.W (Å)				
WT	5.1 ± 0.23	4.8 ± 0.25	5.1 ± 0.27	5.1 ± 0.26
R2 Defect	5.1 ± 0.27	5.1 ± 0.28	5.4 ± 0.30	5.2 ± 0.28
Maj.W (Å)				
WT	11.1 ± 0.30	11.4 ± 0.27	11.5 ± 0.29	11.6 ± 0.29
R1 Defect	11.3 ± 0.30	11.5 ± 0.29	11.3 ± 0.28	11.8 ± 0.33
Maj.D (Å)				
WT	4.6 ± 0.29	4.5 ± 0.31	4.7 ± 0.28	4.6 ± 0.28
R1 Defect	4.3 ± 0.29	4.5 ± 0.32	5.0 ± 0.28	4.7 ± 0.27
R2 Defect	4.5 ± 0.27	4.7 ± 0.31	4.8 ± 0.28	4.5 ± 0.30

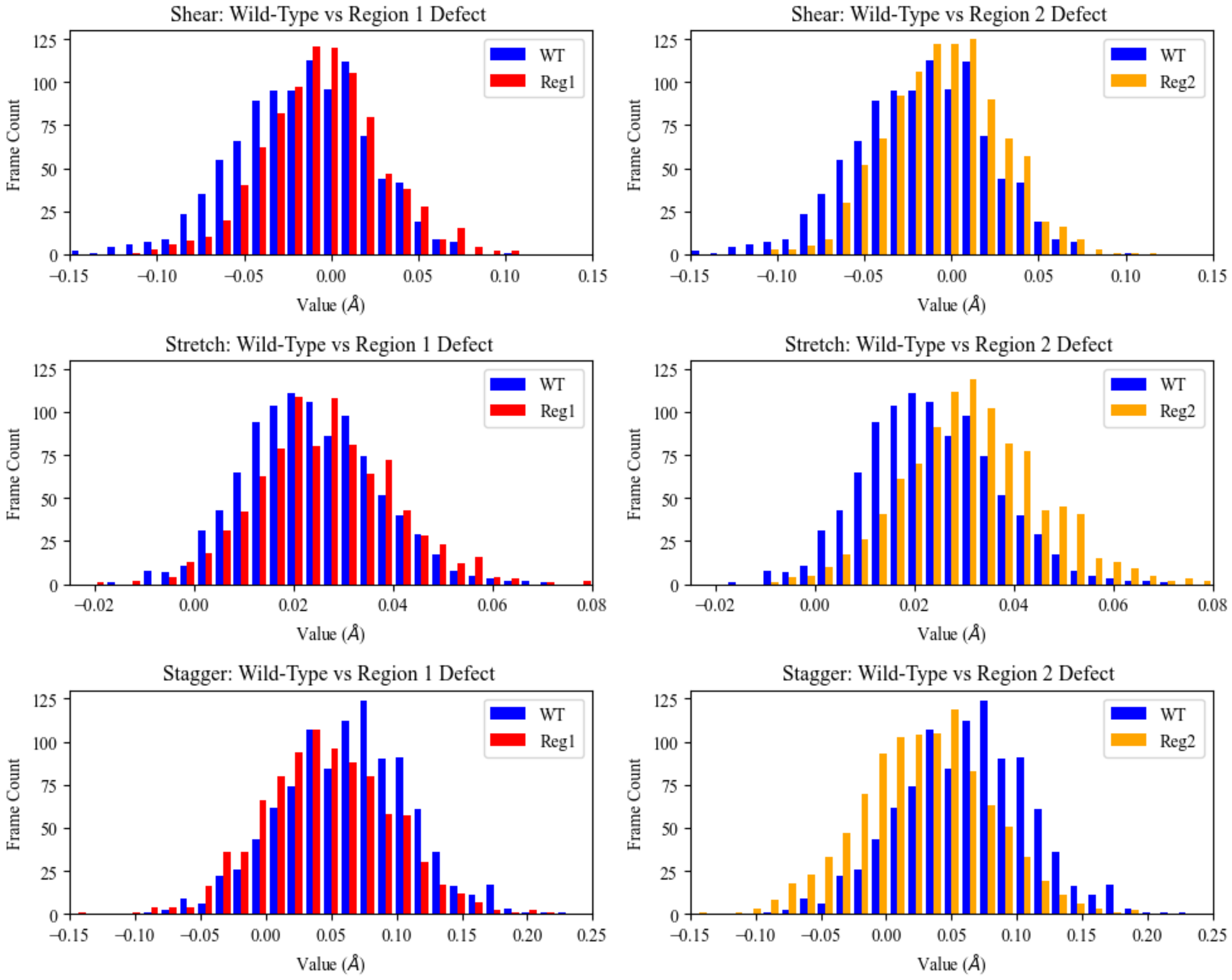

Figure S6: Population distributions of intrabase translational parameters (shear, stretch, and stagger) for wild-type system (WT, blue) vs region 1 defect system (Reg1, red) and for wild-type system (WT, blue) vs region 2 defect system (Reg2, orange). The systems do not include in their analysis the 40 base pairs associated with tail regions.

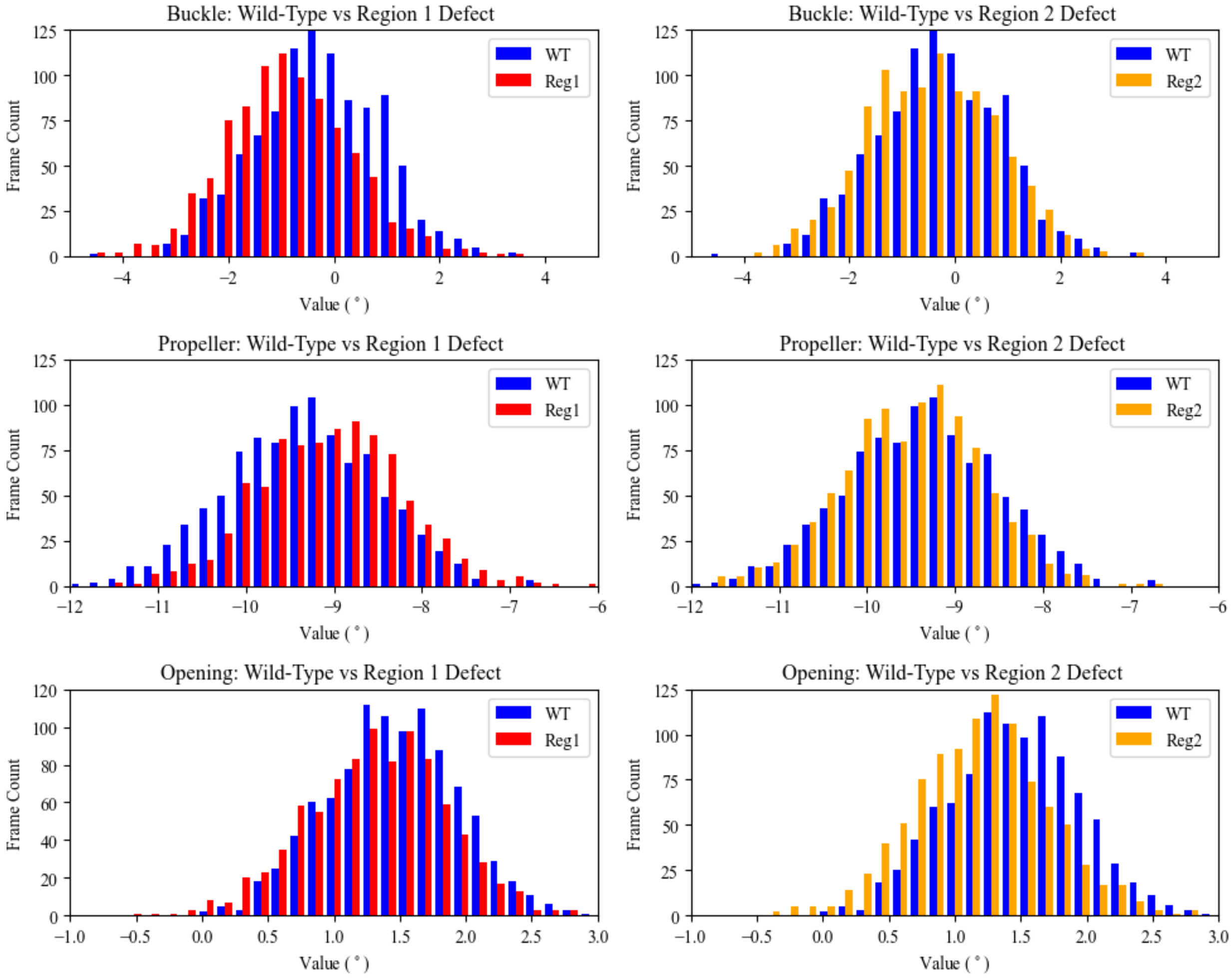

Figure S7: Population distributions of intrabase rotational parameters (buckle, propeller, and opening) for wild-type system (WT, blue) vs region 1 defect system (Reg1, red) and for wild-type (WT, blue) vs region 2 defect system (Reg2, orange). The systems do not include in their analysis the 40 base pairs associated with tail regions.

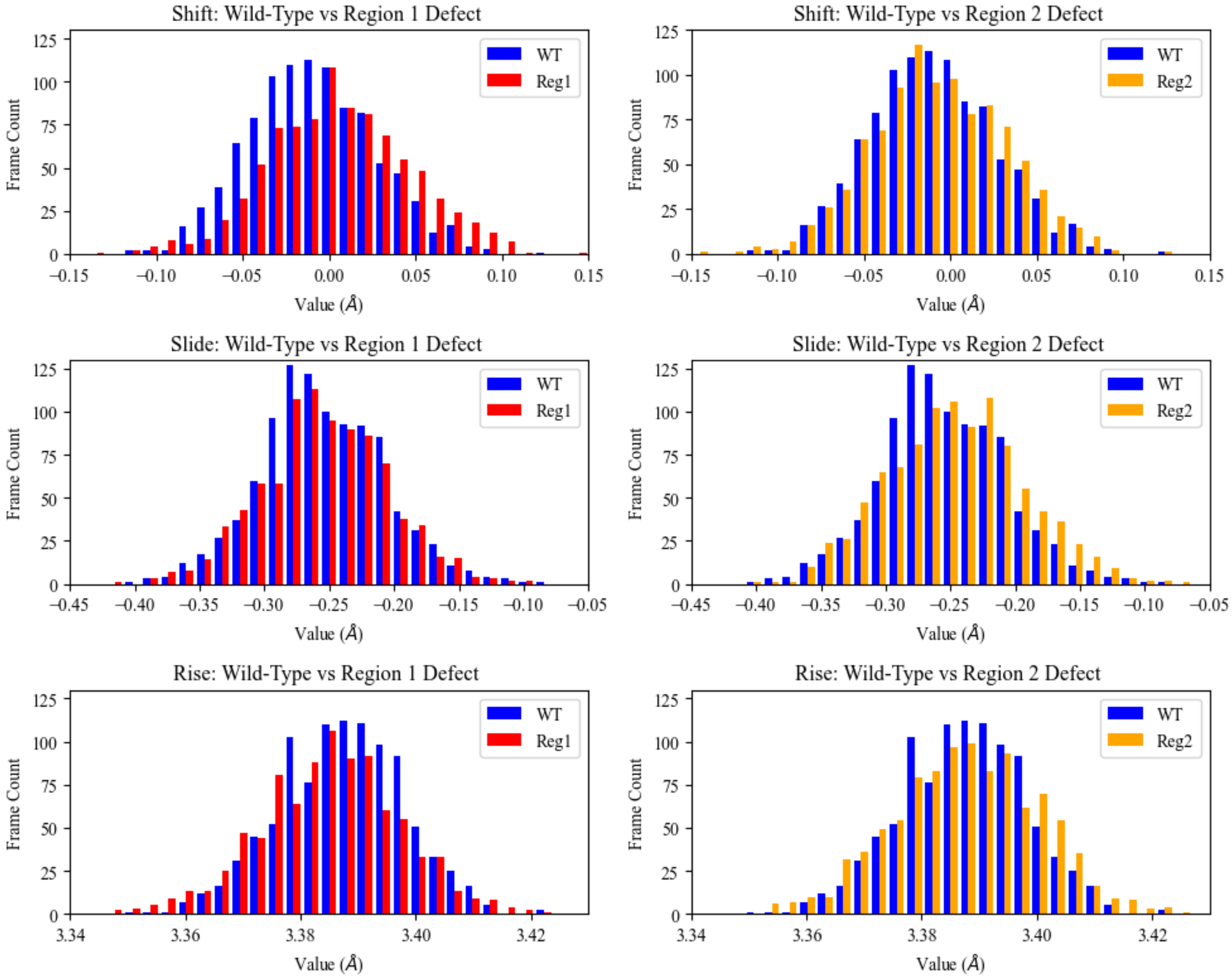

Figure S8: Population distributions of interbase translational parameters (shift, slide, and rise) for wild-type system (WT, blue) vs region 1 defect system (Reg1, red) and for wild-type system (WT, blue) vs region 2 defect system (Reg2, orange). The systems do not include in their analysis the 40 base pairs associated with tail regions.

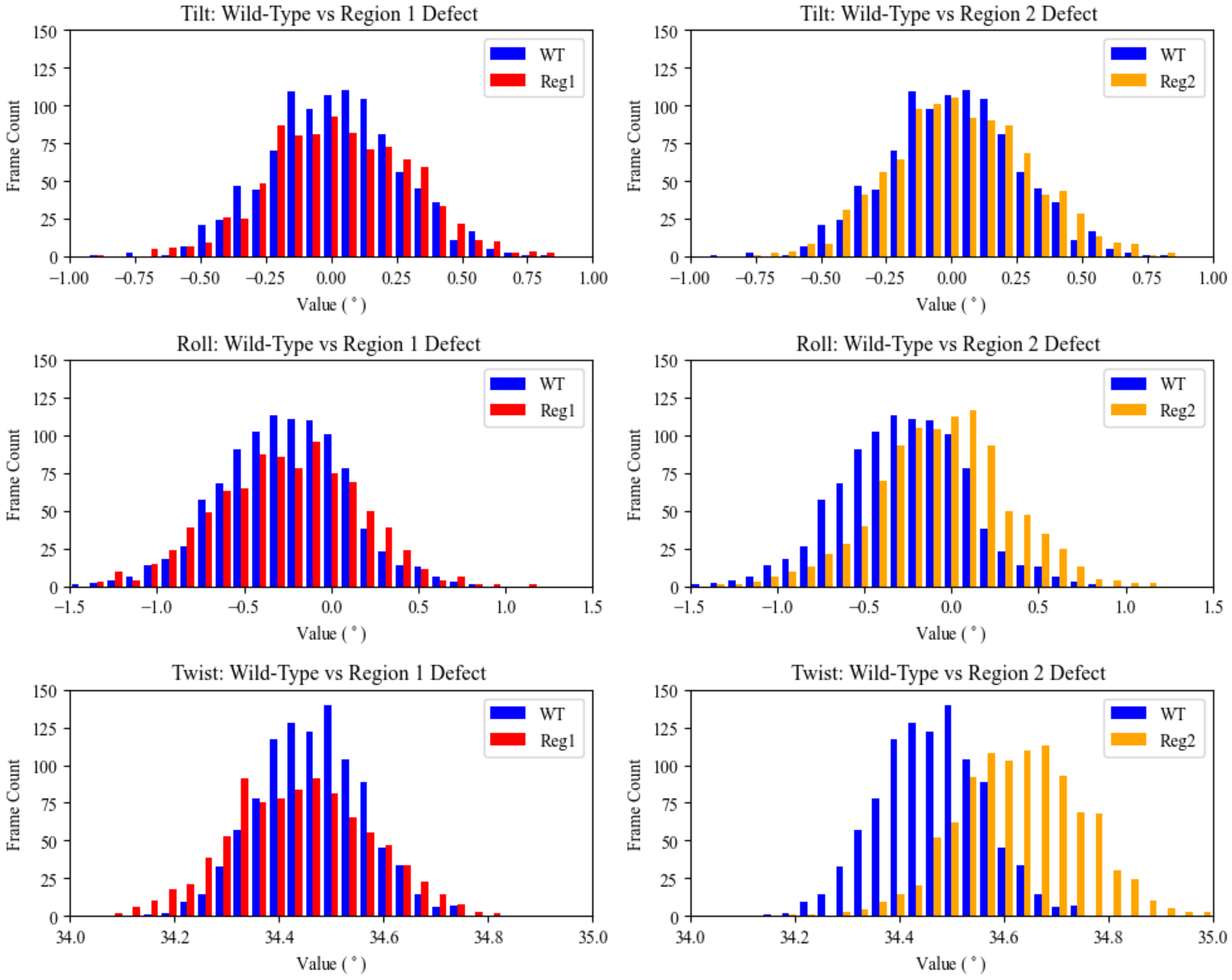

Figure S9: Population distributions of interbase rotational parameters: tilt, roll, and twist for wild-type system (WT, blue) vs region 1 defect system (Reg1, red) and for wild-type system (WT, blue) vs region 2 defect system (Reg2, orange). The systems do not include in their analysis the 40 base pairs associated with tail regions.

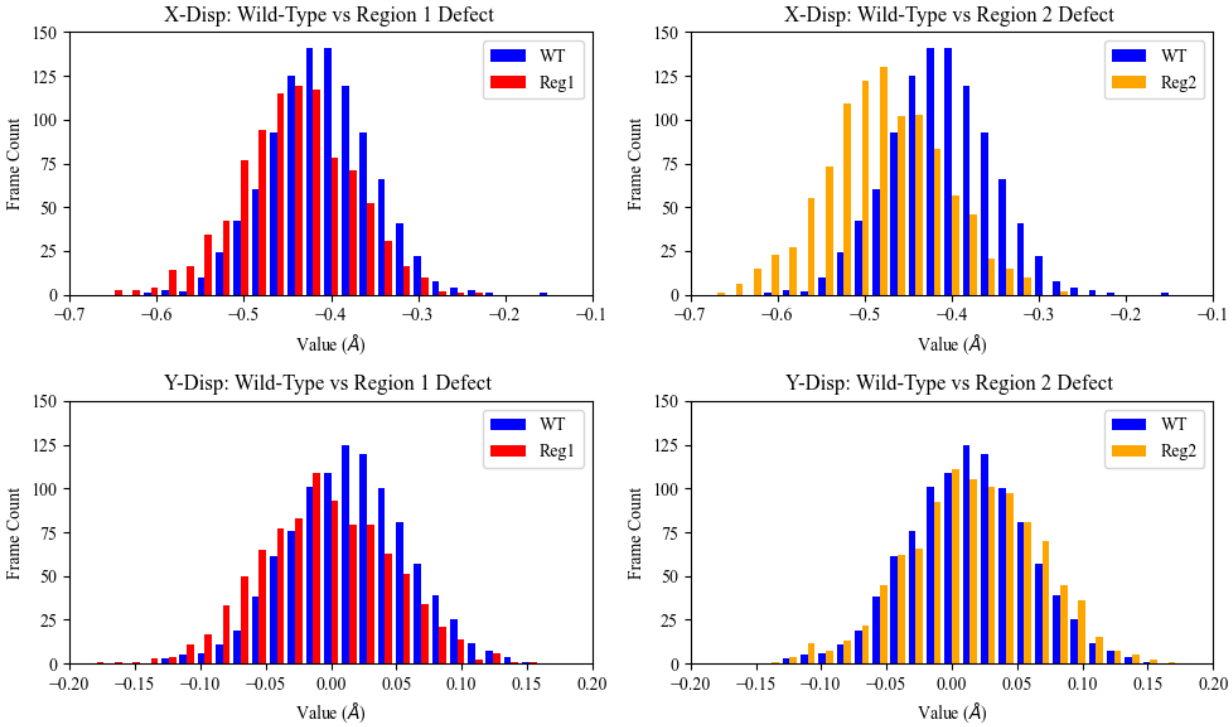

Figure S10: Population distributions of base-axis translational parameters (X- and Y-displacement) for wild-type system (WT, blue) vs region 1 defect system (Reg1, red) and for wild-type system (WT, blue) vs region 2 defect system (Reg2, orange). The systems do not include in their analysis the 40 base pairs associated with tail regions.

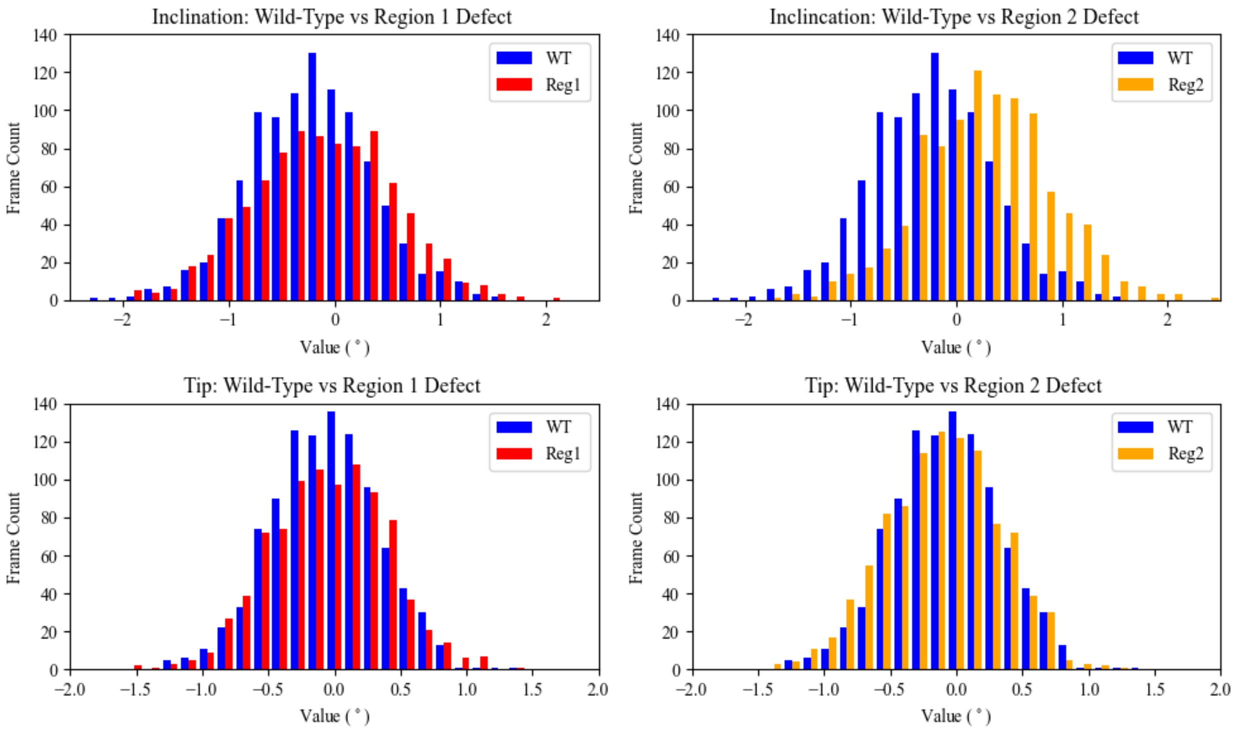

Figure S11: Population distributions of the base-axis rotational parameters (inclination and tip) for wild-type system (WT, blue) vs region 1 defect system (Reg1, red) and for wild-type system (WT, blue) vs region 2 defect system (Reg2, orange). The systems do not include in their analysis the 40 base pairs associated with tail regions.

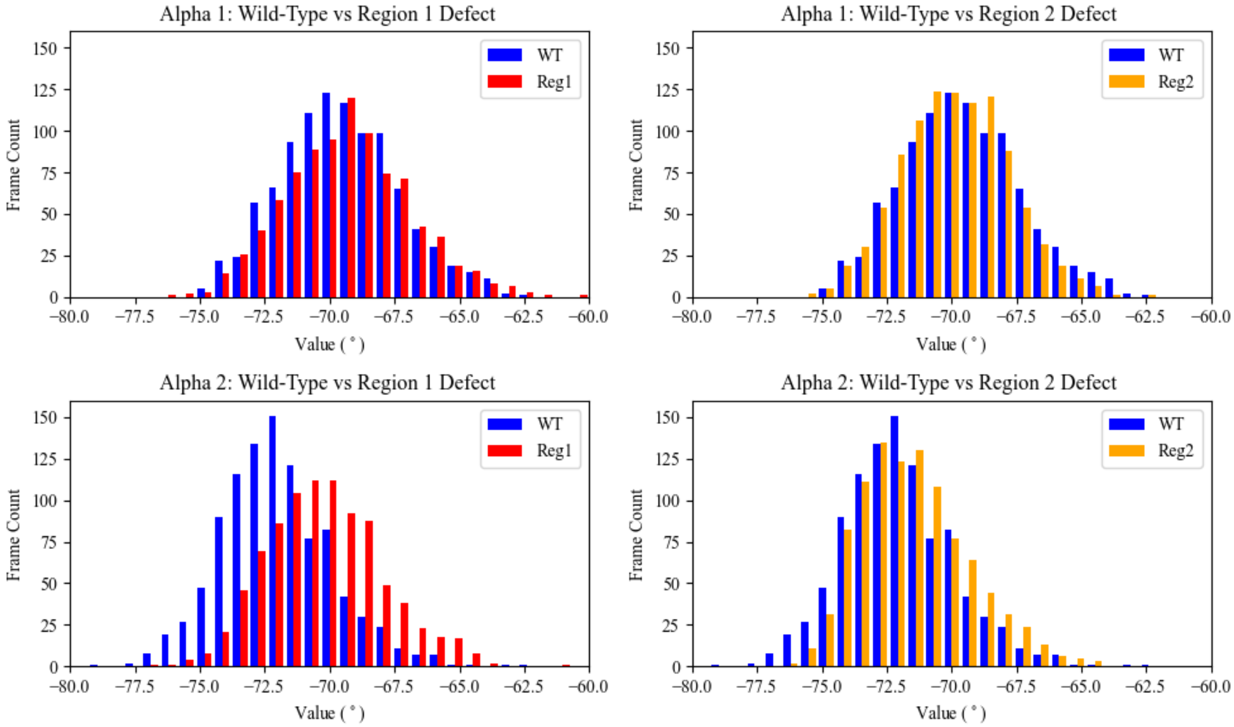

Figure S12: Population distributions of the alpha torsional angle of both DNA strands 1 and 2 for wild-type system (WT, blue) vs region 1 defect system (Reg1, red) and for wild-type system (WT, blue) vs region 2 defect system (Reg2, orange). The systems do not include in their analysis the 40 base pairs associated with tail regions.

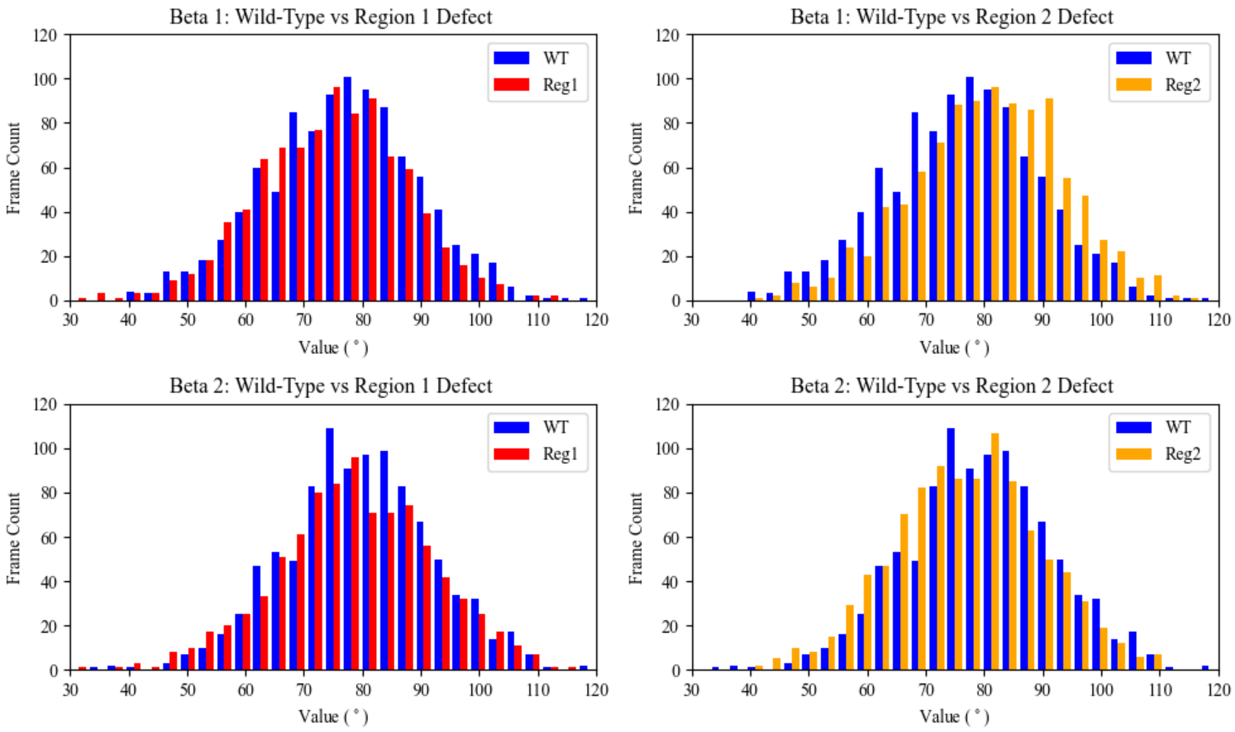

Figure S13: Population distributions of the beta torsional angle of both DNA strands 1 and 2 for wild-type system (WT, blue) vs region 1 defect system (Reg1, red) and for wild-type system (WT, blue) vs region 2 defect system (Reg2, orange). The systems do not include in their analysis the 40 base pairs associated with tail regions.

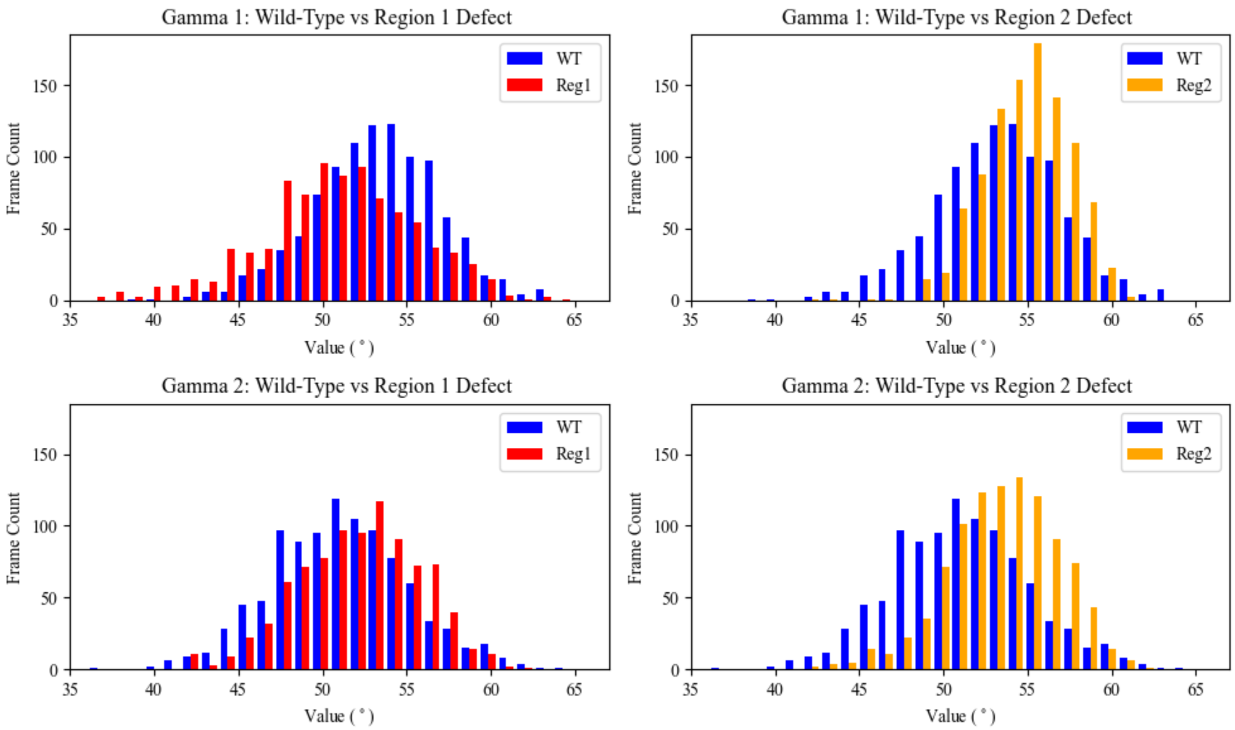

Figure S14: Population distributions of the gamma torsional angle of both DNA strands 1 and 2 for wild-type system (WT, blue) vs region 1 defect system (Reg1, red) and for wild-type system (WT, blue) vs region 2 defect system (Reg2, orange). The systems do not include in their analysis the 40 base pairs associated with tail regions.

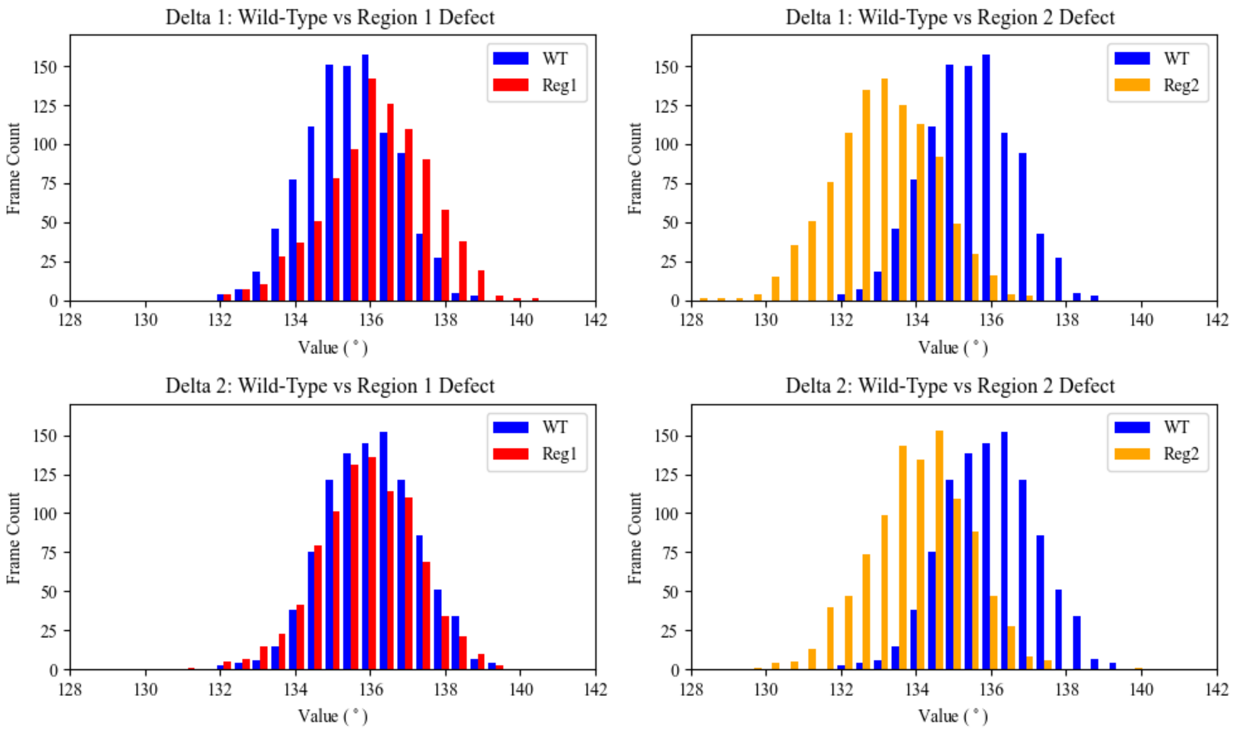

Figure S15: Population distributions of the delta torsional angle of both DNA strands 1 and 2 for wild-type system (WT, blue) vs region 1 defect system (Reg1, red) and for wild-type system (WT, blue) vs region 2 defect system (Reg2, orange). The systems do not include in their analysis the 40 base pairs associated with tail regions.

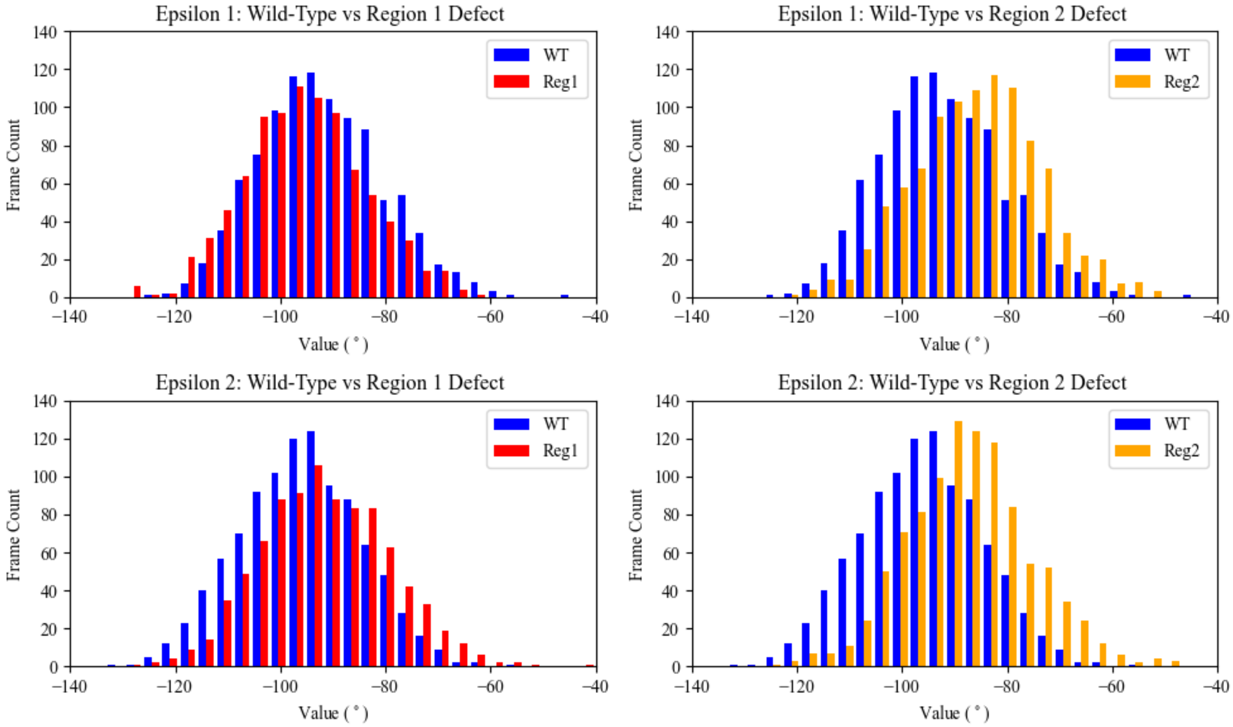

Figure S16: Population distributions of the epsilon torsional angle of both DNA strands 1 and 2 for wild-type system (WT, blue) vs region 1 defect system (Reg1, red) and for wild-type system (WT, blue) vs region 2 defect system (Reg2, orange). The systems do not include in their analysis the 40 base pairs associated with tail regions.

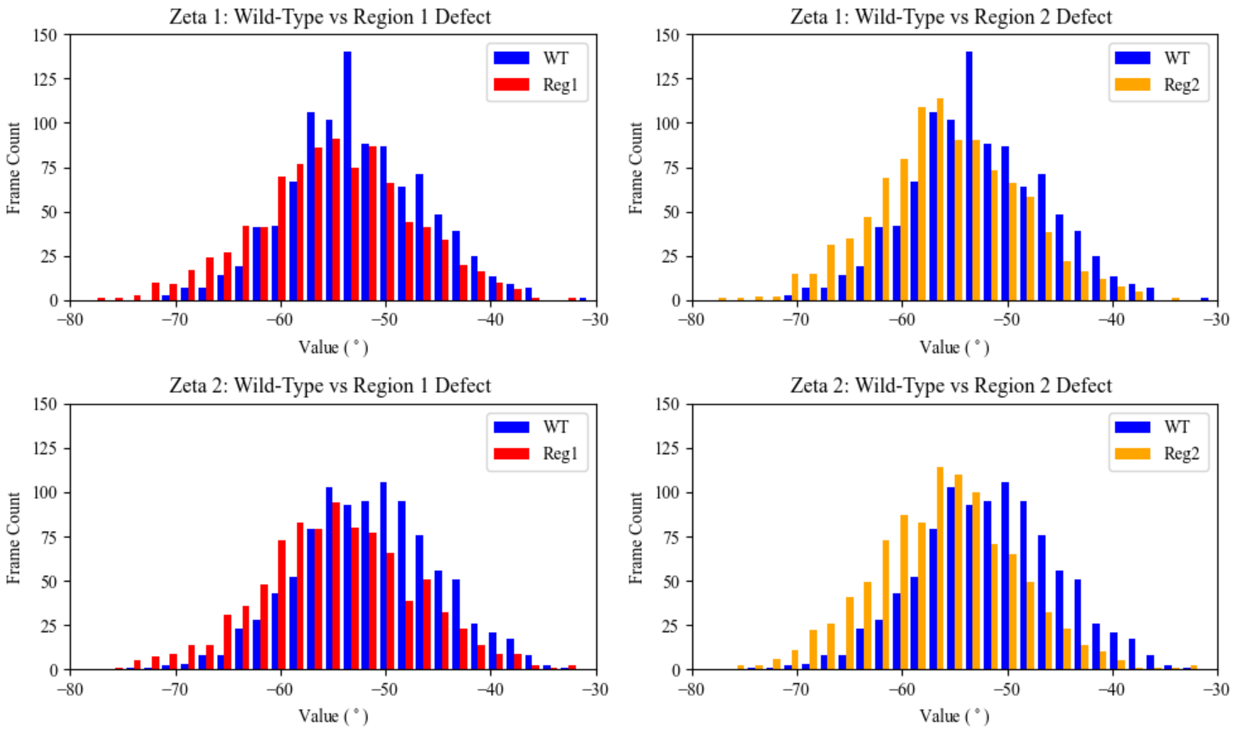

Figure S17: Population distributions of the zeta torsional angle of both DNA strands 1 and 2 for wild-type system (WT, blue) vs region 1 defect system (Reg1, red) and for wild-type system (WT, blue) vs region 2 defect system (Reg2, orange). The systems do not include in their analysis the 40 base pairs associated with tail regions.

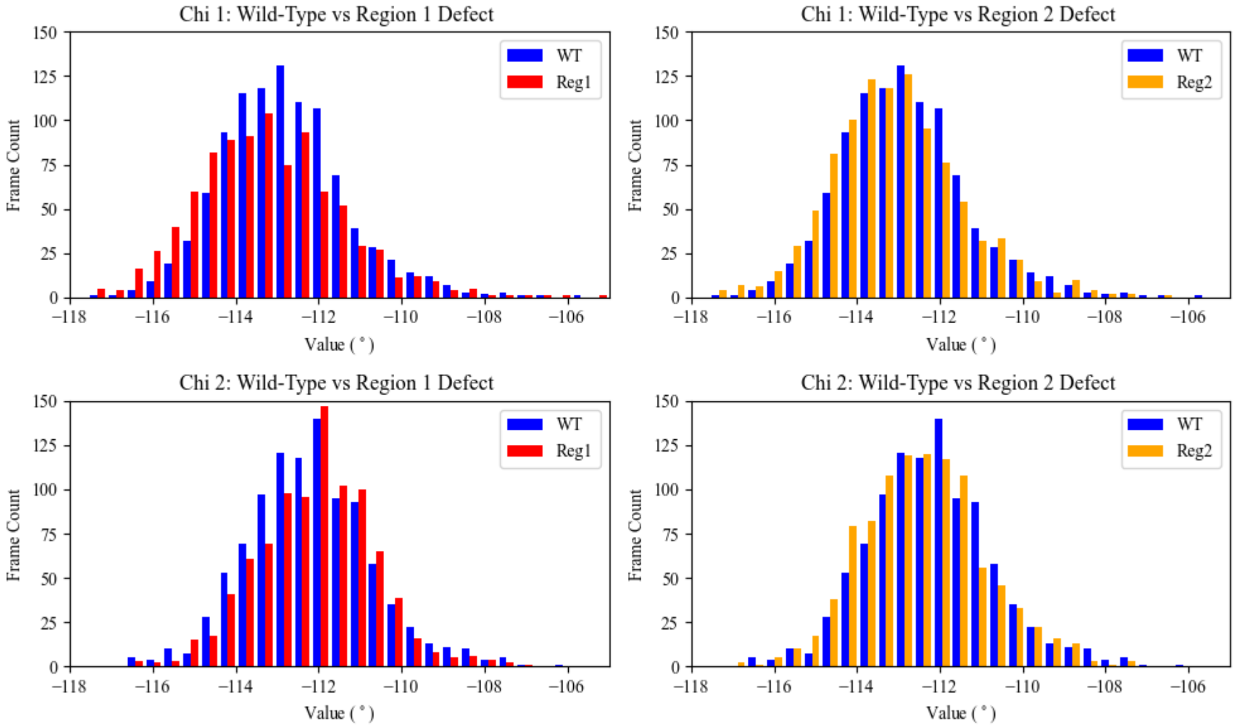

Figure S18: Population distributions of the chi torsional angle of both DNA strands 1 and 2 for wild-type system (WT, blue) vs region 1 defect system (Reg1, red) and for wild-type system (WT, blue) vs region 2 defect system (Reg2, orange). The systems do not include in their analysis the 40 base pairs associated with tail regions.

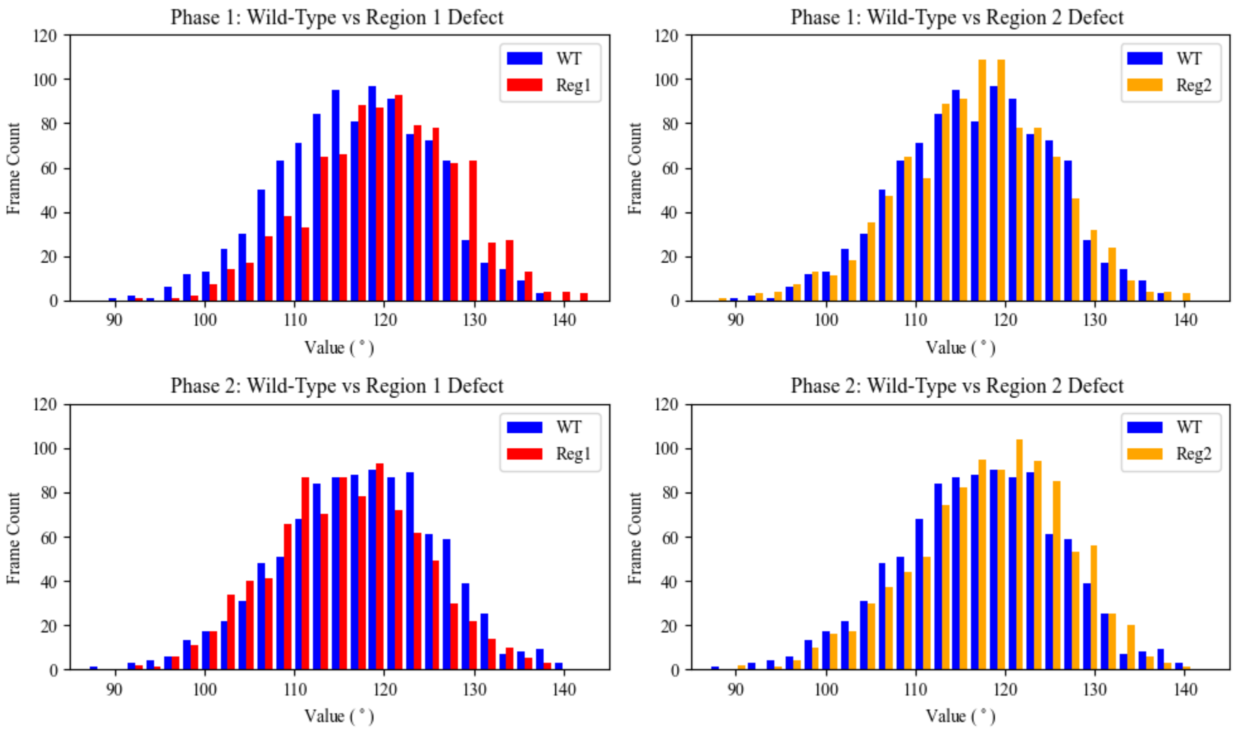

Figure S19: Population distributions of the sugar pucker phase angle of both DNA strands 1 and 2 for wild-type system (WT, blue) vs region 1 defect system (Reg1, red) and for wild-type system (WT, blue) vs region 2 defect system (Reg2, orange). The systems do not include in their analysis the 40 base pairs associated with tail regions.

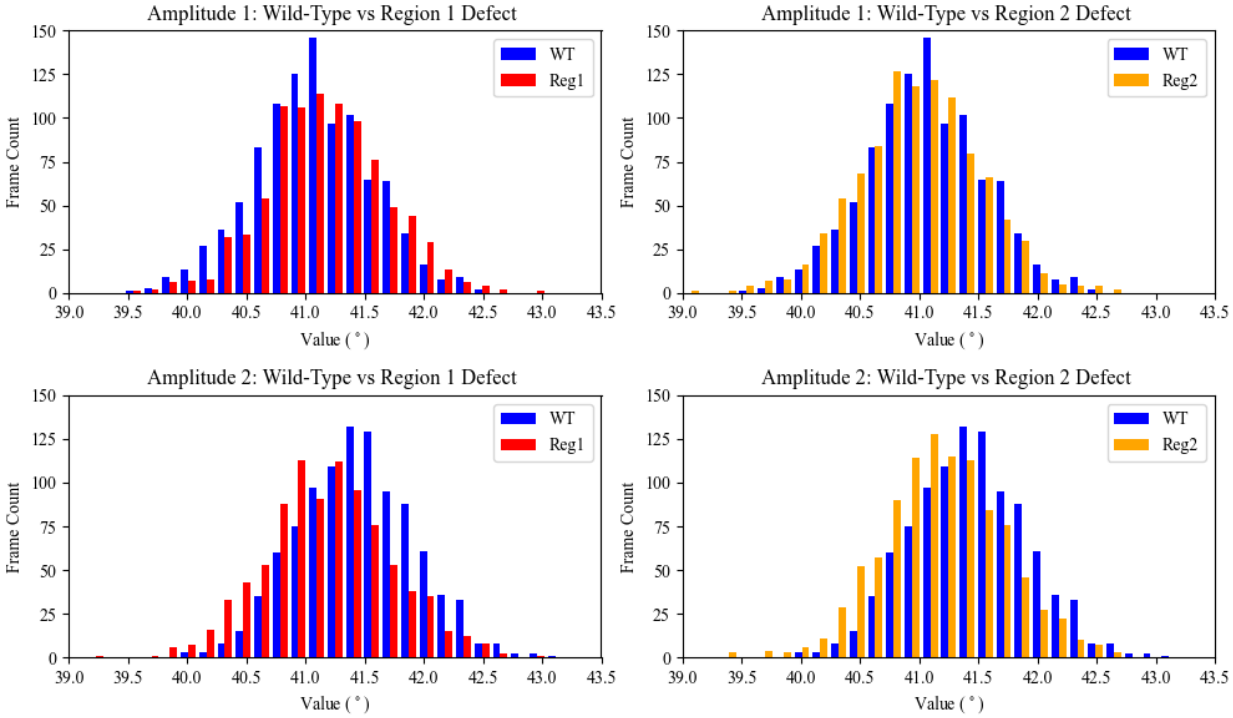

Figure S20: Population distributions of the sugar pucker amplitude of both DNA strands 1 and 2 for wild-type system (WT, blue) vs region 1 defect system (Reg1, red) and for wild-type system (WT, blue) vs region 2 defect system (Reg2, orange). The systems do not include in their analysis the 40 base pairs associated with tail regions.

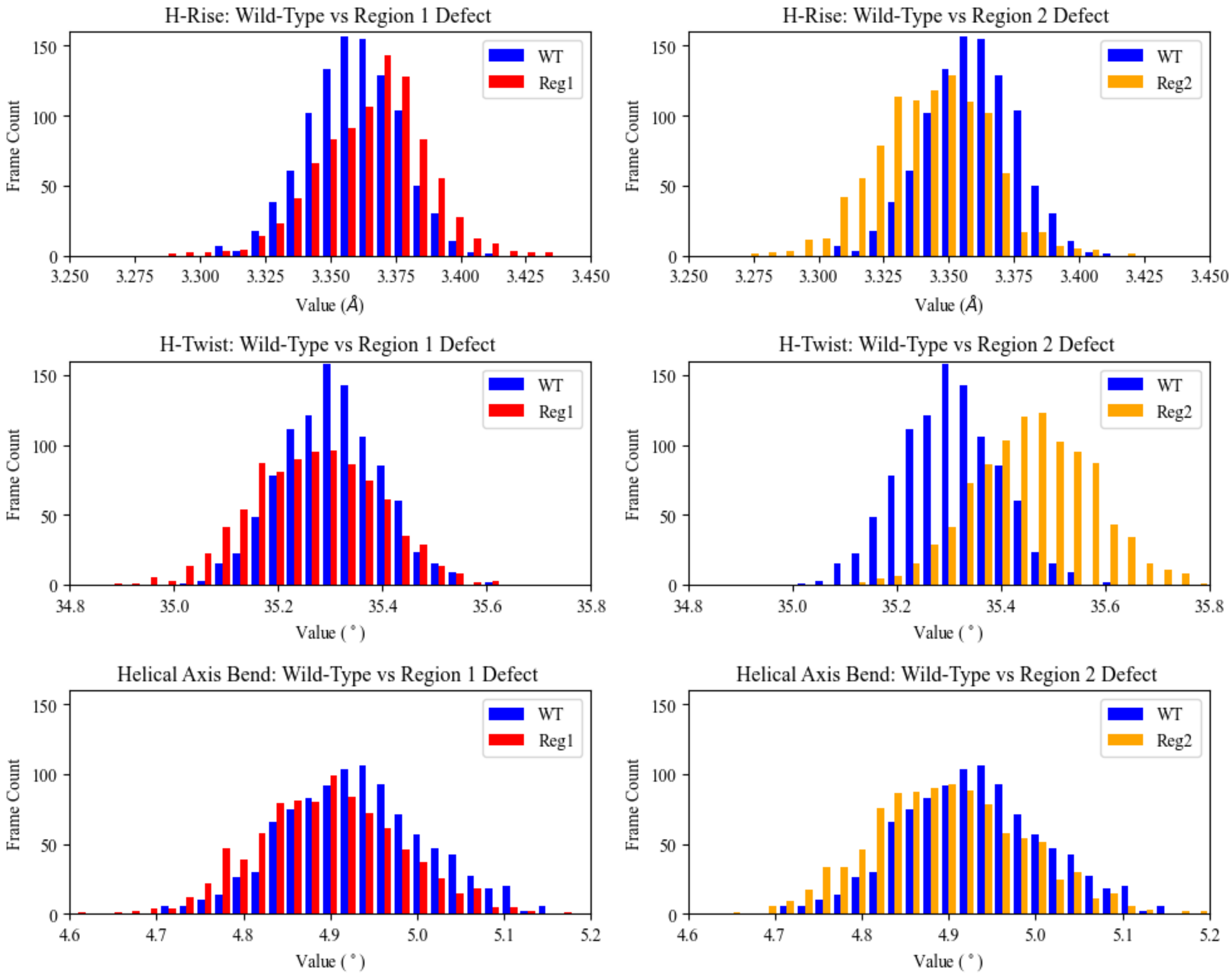

Figure S21: Population distributions of the helical rise, helical twist and the helical axis bend for wild-type system (WT, blue) vs region 1 defect system (Reg1, red) and for wild-type system (WT, blue) vs region 2 defect system (Reg2, orange). The systems do not include in their analysis the 40 base pairs associated with tail regions.

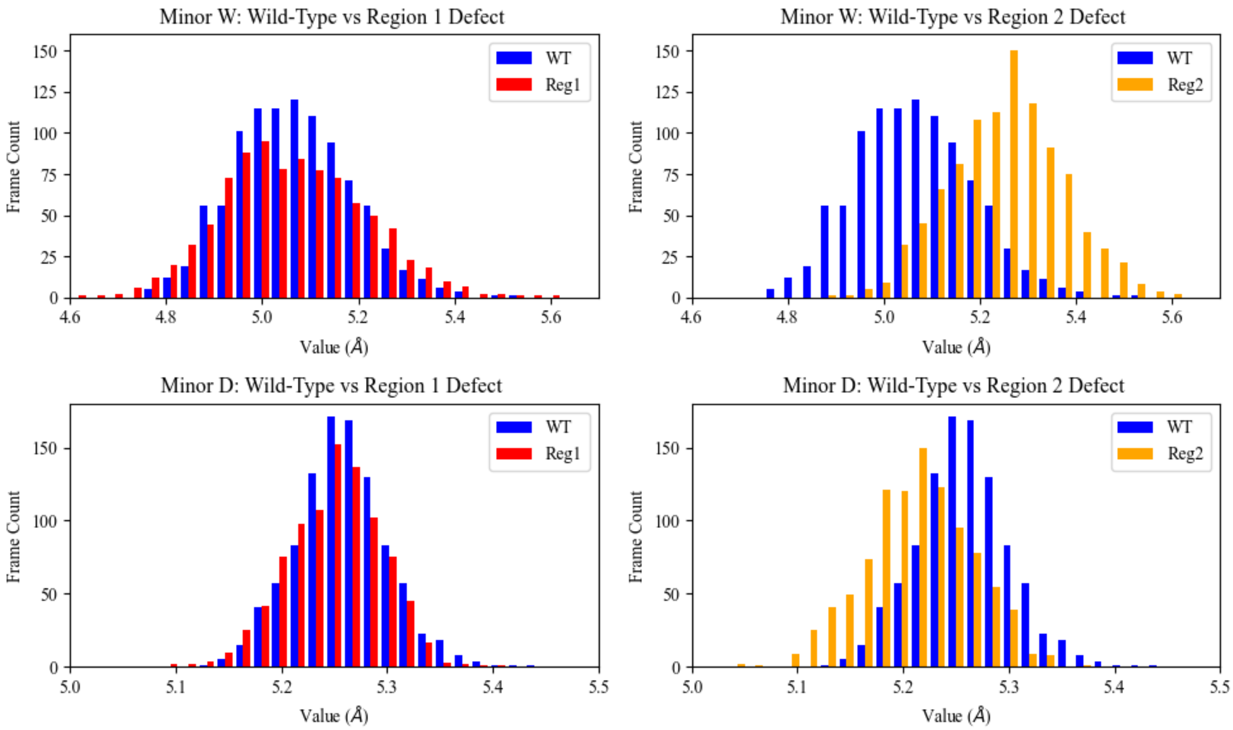

Figure S22: Population distributions of the minor groove width and depth for wild-type system (WT, blue) vs region 1 defect system (Reg1, red) and for wild-type system (WT, blue) vs region 2 defect system (Reg2, orange). The systems do not include in their analysis the 40 base pairs associated with tail regions.

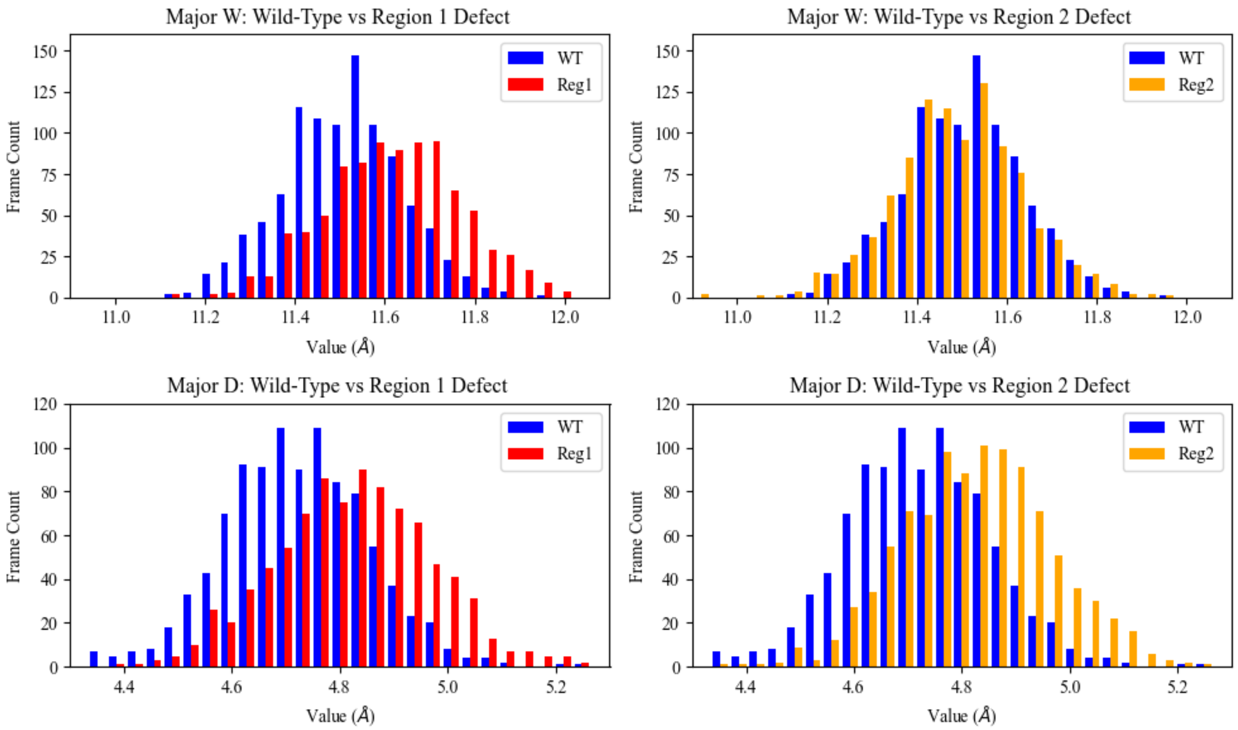

Figure S23: Population distributions of the major groove width and depth for wild-type system (WT, blue) vs region 1 defect system (Reg1, red) and for wild-type system (WT, blue) vs region 2 defect system (Reg2, orange). The systems do not include in their analysis the 40 base pairs associated with tail regions.

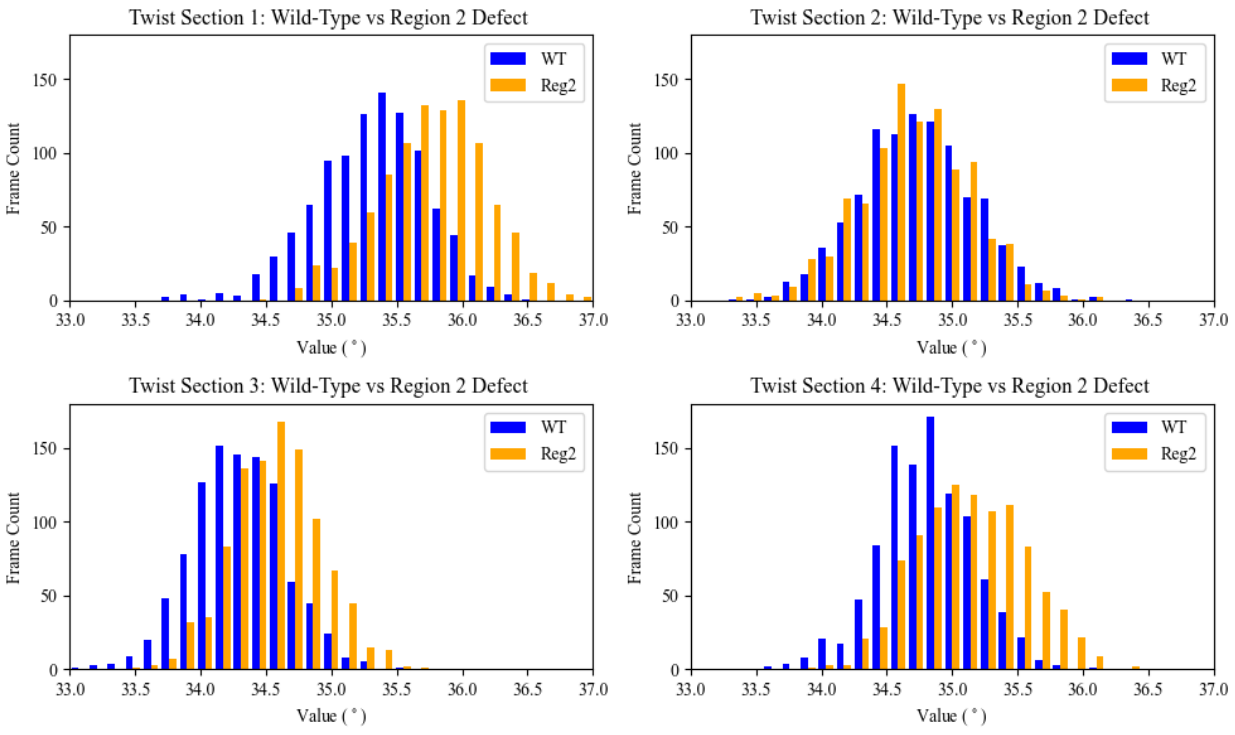

Figure S24: Population distributions of the twist parameter by NCP section for wild-type system (WT, blue) vs region 2 defect system (Reg2, orange).

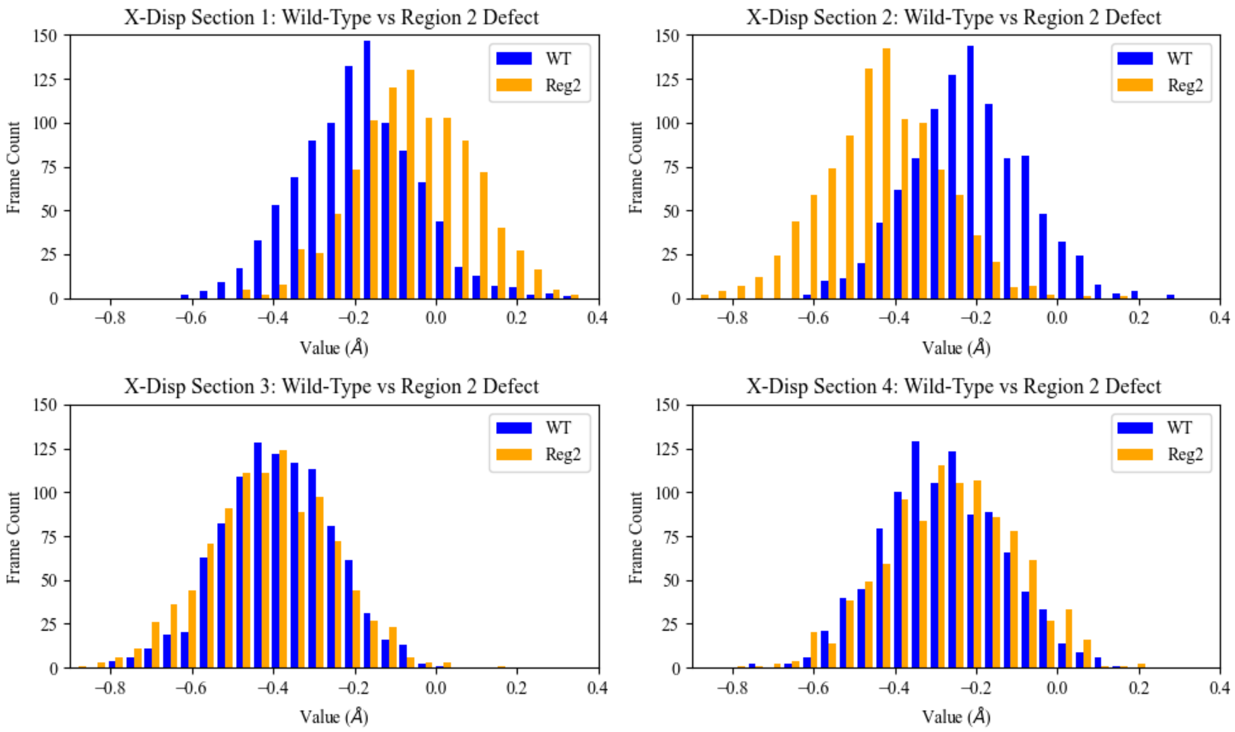

Figure S25: Population distributions of the x-displacement parameter by NCP section for wild-type system (WT, blue) vs region 2 defect system (Reg2, orange).

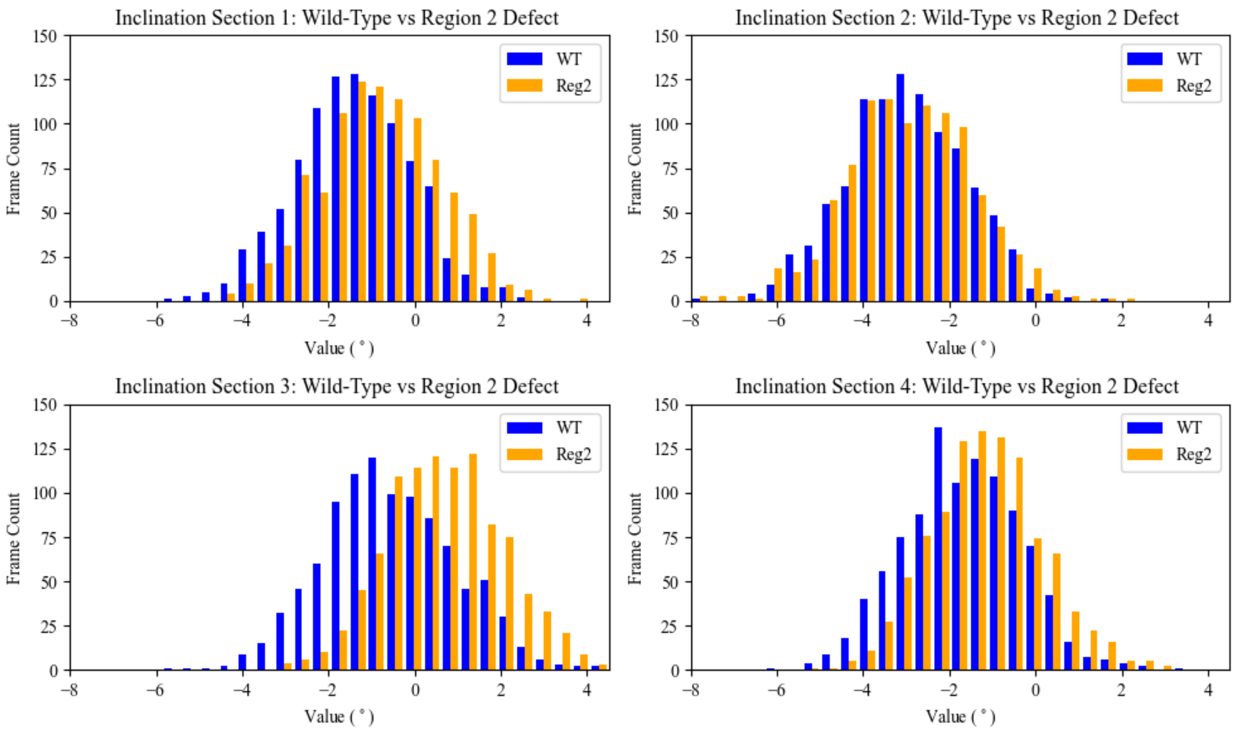

Figure S26: Population distributions of the inclination parameter by NCP section for wild-type system (WT, blue) vs region 2 defect system (Reg2, orange).

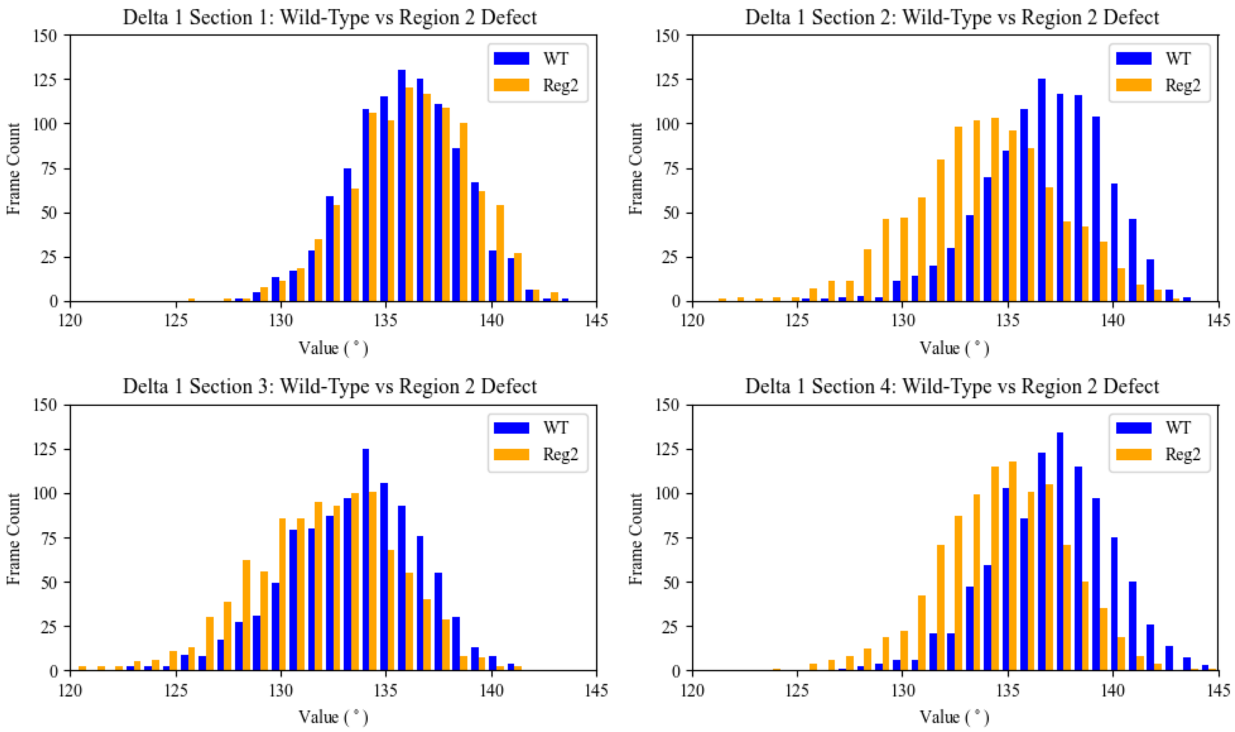

Figure S27: Population distributions of the delta torsional angle on strand 1 by NCP section for wild-type system (WT, blue) vs region 2 defect system (Reg2, orange).

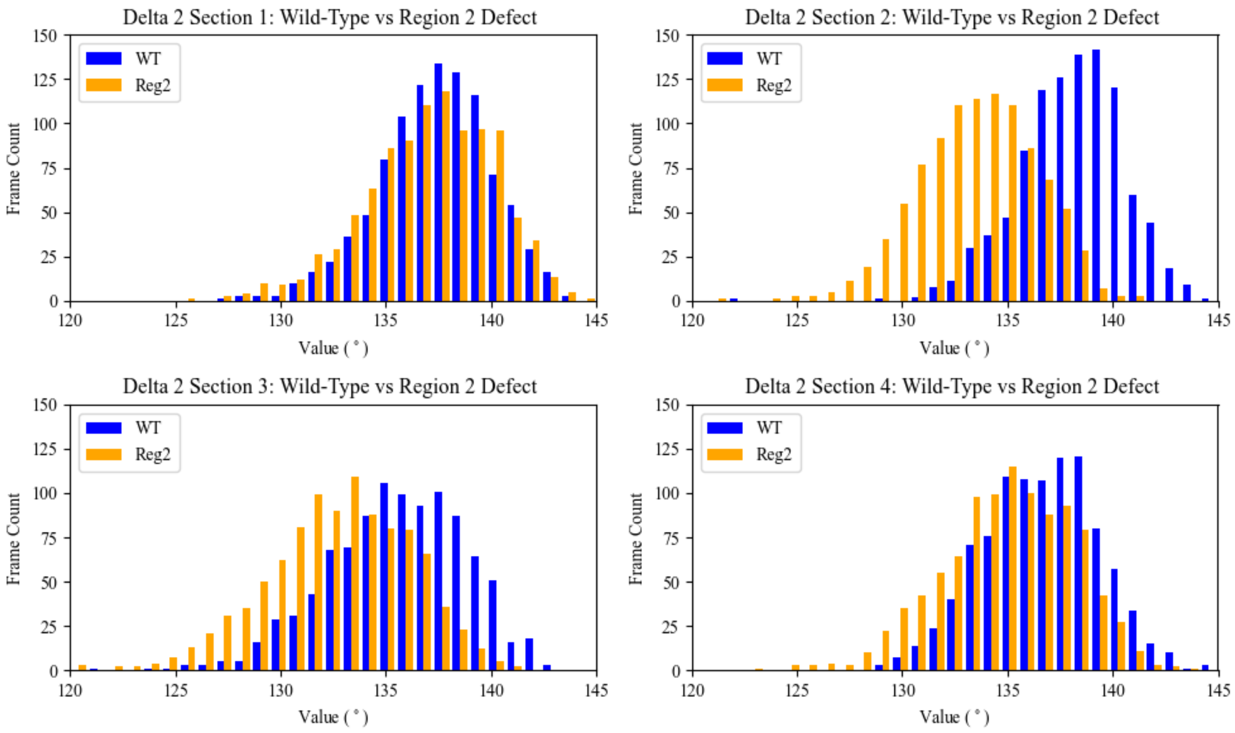

Figure S28: Population distributions of the delta torsional angle on strand 2 by NCP section for wild-type system (WT, blue) vs region 2 defect system (Reg2, orange).

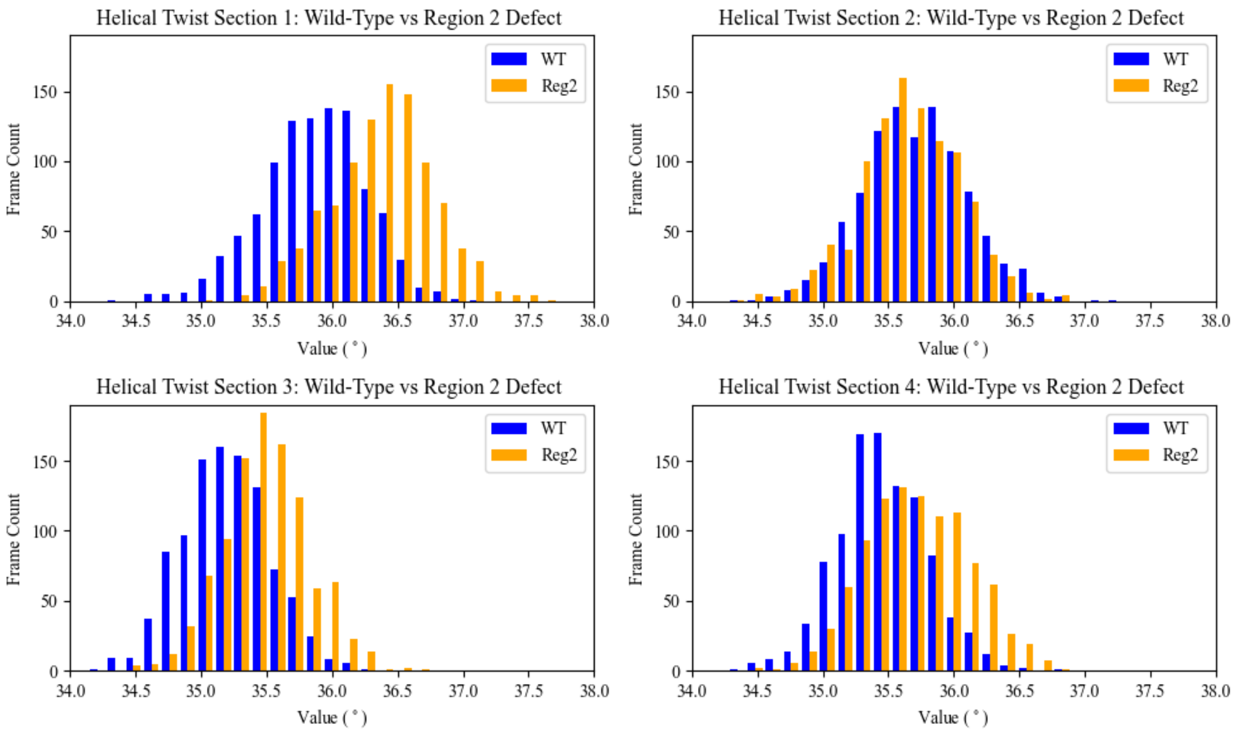

Figure S29: Population distributions of the helical twist parameter by NCP section for wild-type system (WT, blue) vs region 2 defect system (Reg2, orange).

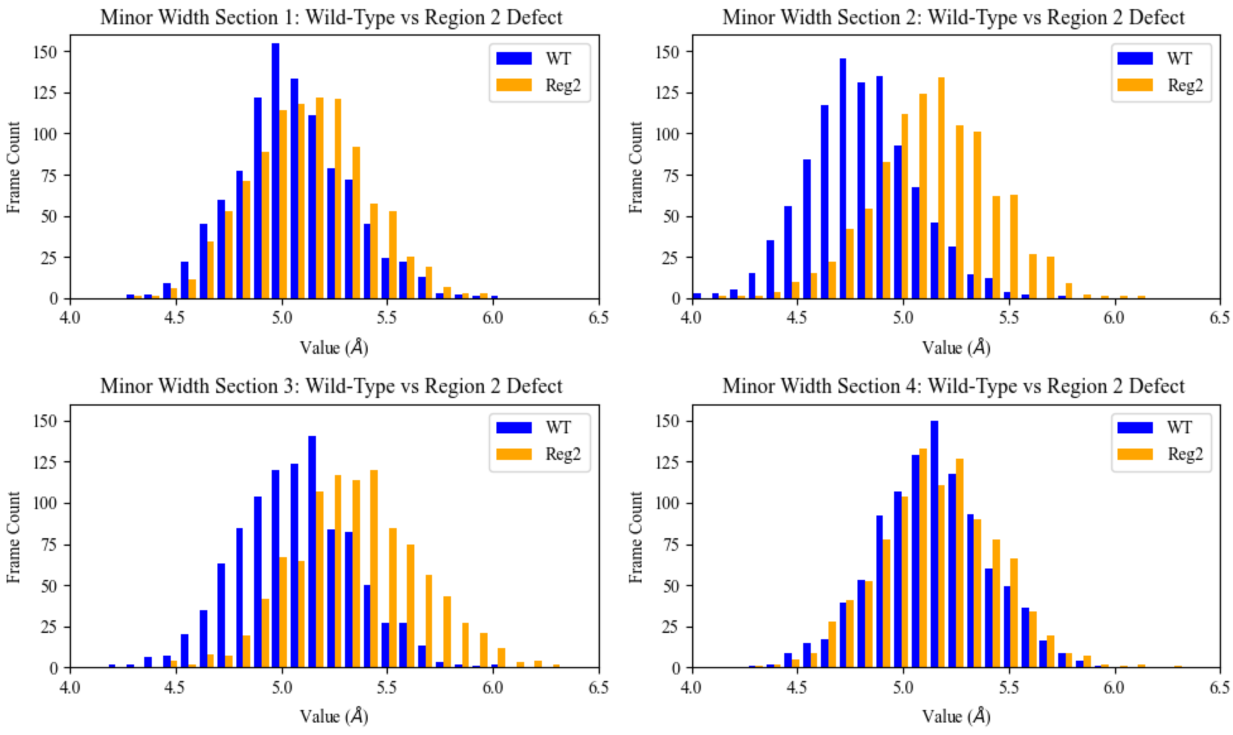

Figure S30: Population distributions of the minor groove width by NCP section for wild-type system (WT, blue) vs region 2 defect system (Reg2, orange).

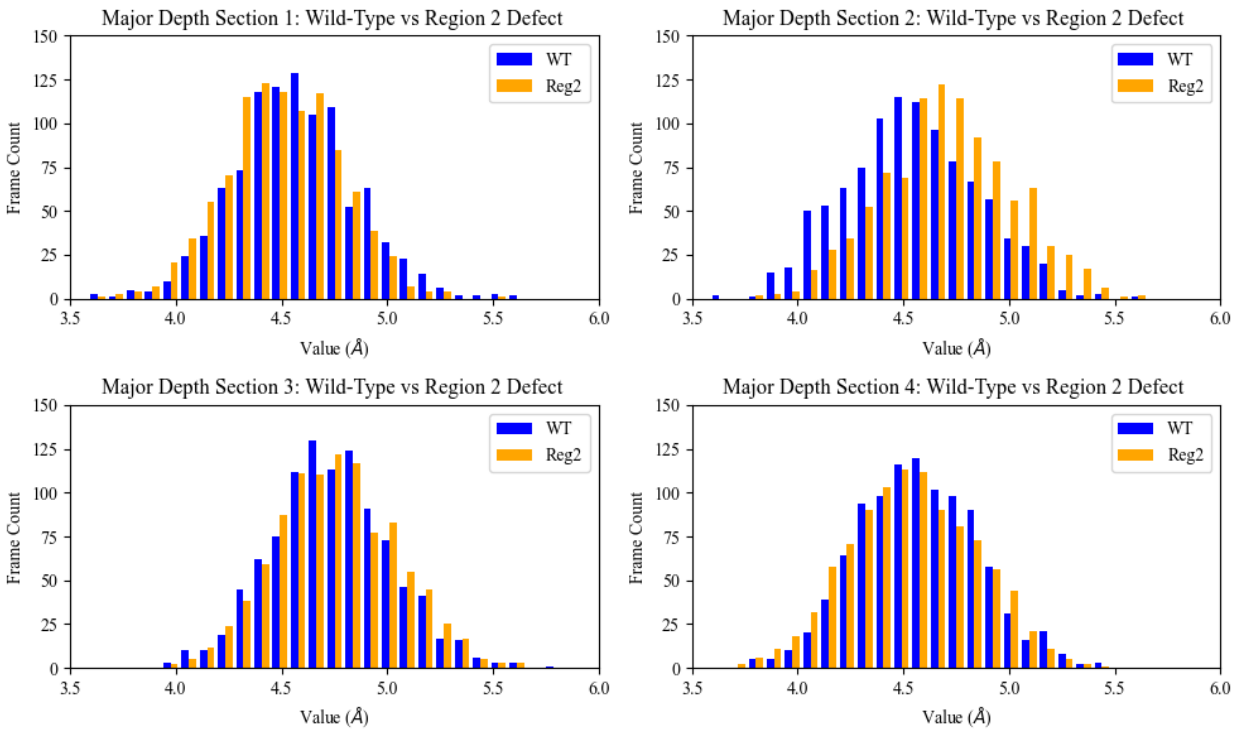

Figure S31: Population distributions of the major groove depth by NCP section for wild-type system (WT, blue) vs region 2 defect system (Reg2, orange).

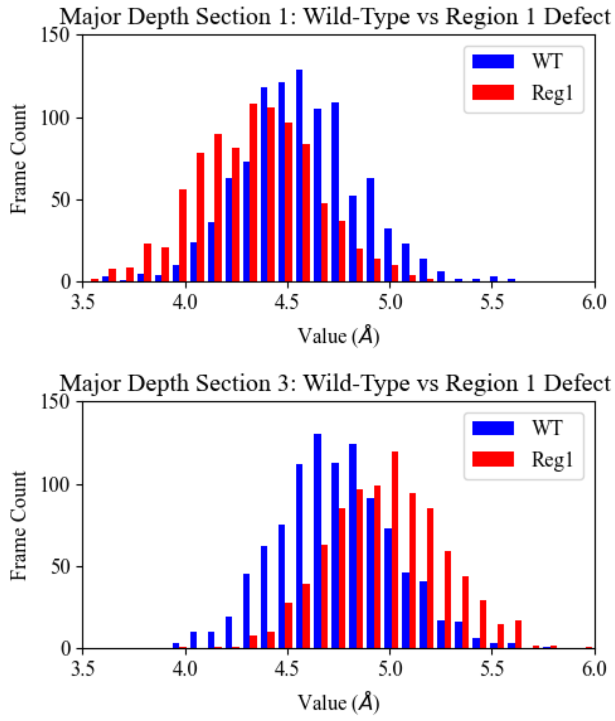

Figure S32: Population distributions of the major groove depth by NCP section for wild-type system (WT, blue) vs region 1 defect system (Reg1, red).

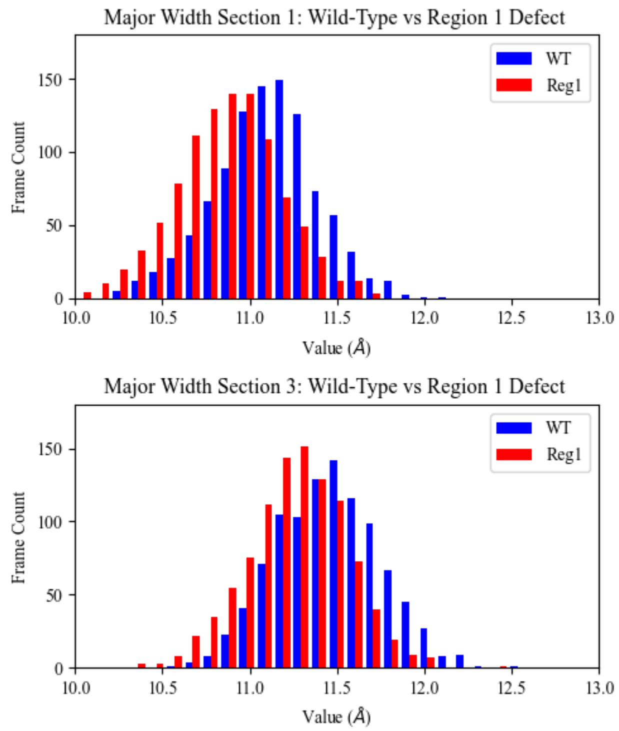

Figure S33: Population distributions of the major groove width by NCP section for wild-type system (WT, blue) vs region 1 defect system (Reg1, red).

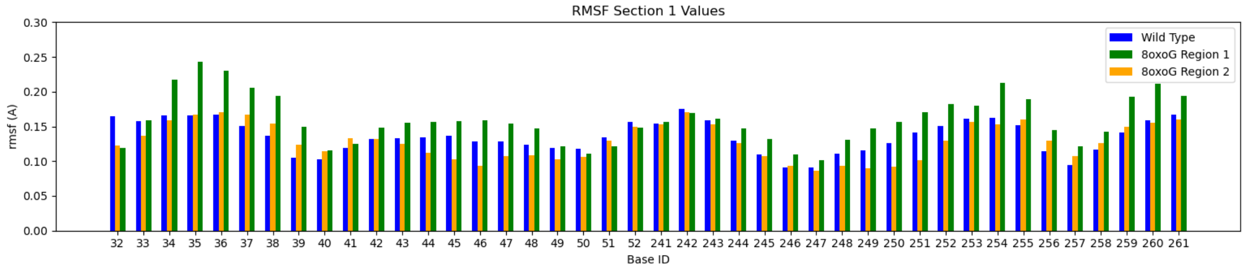

Figure S34: RMSF evaluation of section 1 bases for wild-type system (blue) vs region 1 defect system (green) and vs region 2 defect system (orange).

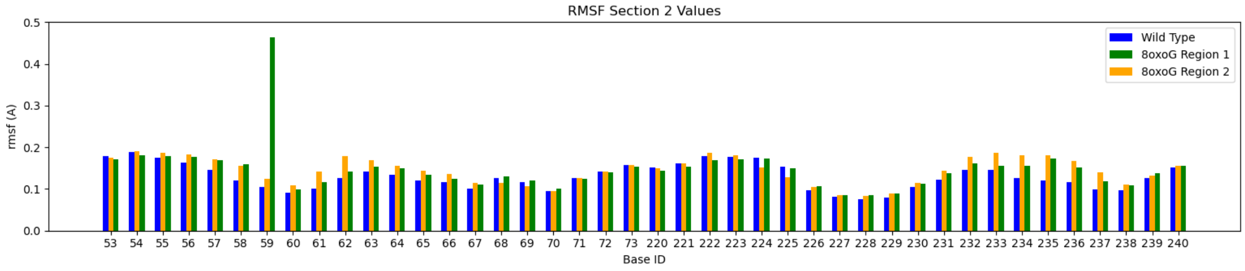

Figure S35: RMSF evaluation of section 2 bases for wild-type system (blue) vs region 1 defect system (green) and vs region 2 defect system (orange).Base number 59 of region 1 is the defect base.

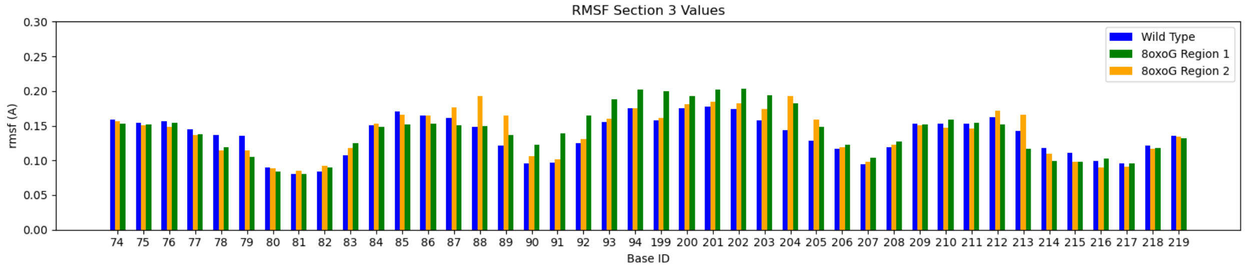

Figure S36: RMSF evaluation of section 3 bases for wild-type system (blue) vs region 1 defect system (green) and vs region 2 defect system (orange).Base number 205 of region 2 is the defect base.

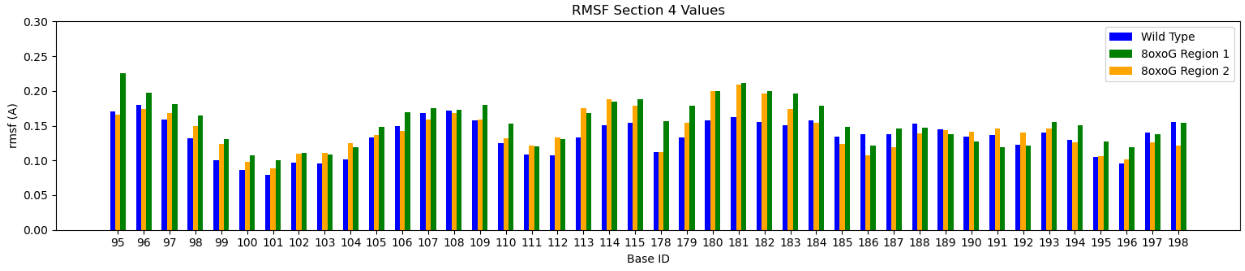

Figure S37: RMSF evaluation of section 4 bases for wild-type system (blue) vs region 1 defect system (green) and vs region 2 defect system (orange).

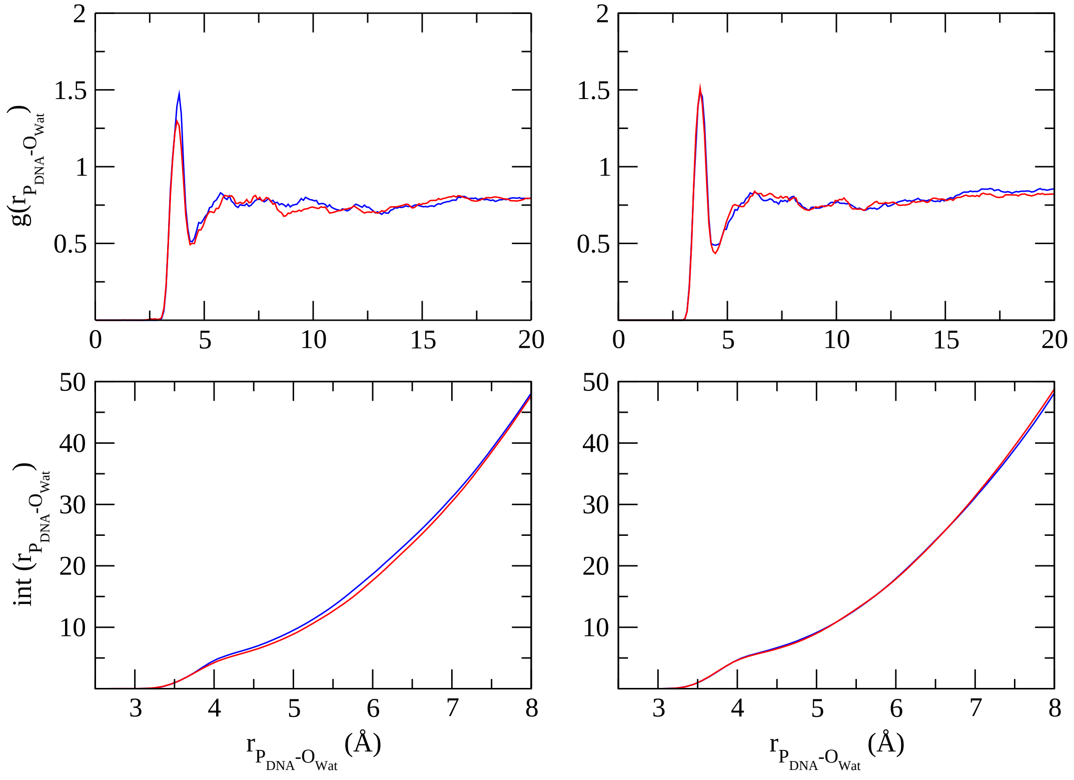

Figure S38: QM DNA-water radial distribution functions g(r$_{P-O_{wat}}$), (top) and integrals (bottom) for the P and O_{wat} atoms, for the reduced state (blue colour) and oxidized state (red colour) of the native (left) and defect system (right) of **region 1**.

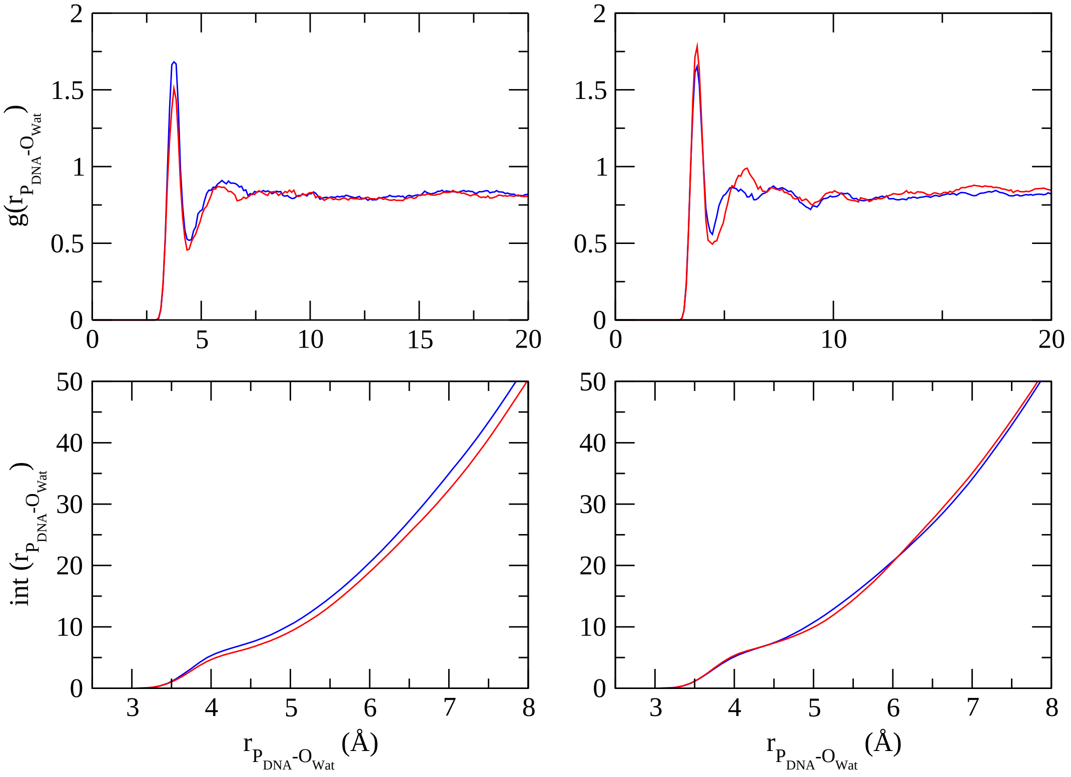

Figure S39: QM DNA-water radial distribution functions g($r_{P-O_{wat}}$), (top) and integrals (bottom) for the P and O_{wat} atoms, for the reduced state (blue colour) and oxidized state (red colour) of the native (left) and defect system (right) of **region 2**.

Author Contributions

† Murat Kılıç and Polydefkis Diamantis contributed equally to the presented work and the preparation of this manuscript.
